# Synthesis of Antiprotozoal 2-(4-Alkyloxyphenyl)-Imidazolines and Imidazoles and Their Evaluation on *Leishmania mexicana* and *Trypanosoma cruzi*

**DOI:** 10.3390/ijms25073673

**Published:** 2024-03-26

**Authors:** Jenifer Torres-Jaramillo, René Blöcher, Karla Fabiola Chacón-Vargas, Jorge Hernández-Calderón, Luvia E. Sánchez-Torres, Benjamín Nogueda-Torres, Alicia Reyes-Arellano

**Affiliations:** 1 Departamento de Química Orgánica, Escuela Nacional de Ciencias Biológicas del Instituto Politécnico Nacional (ENCB-IPN), Mexico City 11340, Mexico; jenifer.t.j@hotmail.com (J.T.-J.); rene.bloecher@googlemail.com (R.B.); jahernandezcal@ipn.mx (J.H.-C.); 2 Facultad de Ciencias Químicas, Universidad Autónoma de Chihuahua, Chihuahua 31100, Mexico; kchacon@uach.mx; 3 Departamento de Inmunología, Escuela Nacional de Ciencias Biológicas del Instituto Politécnico Nacional (ENCB-IPN), Mexico City 11340, Mexico; 4 Departamento de Parasitología, Escuela Nacional de Ciencias Biológicas del Instituto Politécnico Nacional (ENCB-IPN), Mexico City 11340, Mexico; bnogueda@ipn.mx

**Keywords:** imidazoline synthesis, imidazole synthesis, Chagas disease, *Leishmania mexicana*, *Trypanosoma cruzi*

## Abstract

Twenty 2-(4-alkyloxyphenyl)-imidazolines and 2-(4-alkyloxyphenyl)-imidazoles were synthesized, with the former being synthesized in two steps by using MW and ultrasonication energy, resulting in good to excellent yields. Imidazoles were obtained in moderate yields by oxidizing imidazolines with MnO_2_ and MW energy. In response to the urgent need to treat neglected tropical diseases, a set of 2-(4-alkyloxyphenyl)- imidazolines and imidazoles was tested in vitro on *Leishmania mexicana* and *Trypanosoma cruzi*. The leishmanicidal activity of ten compounds was evaluated, showing an IC_50_ < 10 µg/mL. Among these compounds, **27**–**31** were the most active, with IC_50_ values < 1 µg/mL (similar to the reference drugs). In the evaluation on epimastigotes of *T. cruzi*, only **30** and **36** reached an IC_50_ < 1 µg/mL, showing better inhibition than both reference drugs. However, compounds **29**, **33**, and **35** also demonstrated attractive trypanocidal activities, with IC_50_ values < 10 µg/mL, similar to the values for benznidazole and nifurtimox.

## 1. Introduction

According to the World Health Organization (WHO), more than one billion people around the world suffer from neglected tropical diseases, which include Leishmaniasis and Chagas disease. Leishmaniasis affects ~12 million people worldwide, with ~1–2 million new cases per year. Chagas disease is mostly found in Latin America, where it threatens the lives of 8–10 million people [1].

The etiologic agent of leishmaniasis is *Leishmania* spp. [2,3], a vector-born flagellate protozoan parasite. In the American continents, it is transmitted to humans from other vertebrate hosts by *Lutzomyia* spp. sand flies [3]. The disease has three principal forms: cutaneous leishmaniasis (characterized by a localized ulcerative lesion or diffuse nodular lesions), mucocutaneous leishmaniasis (manifesting in the destruction of oral mucosal tissue), and visceral leishmaniasis (a life-threatening condition involving severe organ damage). This disease depends mainly on the infecting species. For example, an infection with *Leishmania mexicana* tends to trigger either localized or diffuse cutaneous leishmaniasis [4].

Chagas disease, caused by the hemoflagellate protozoan *Trypanosoma cruzi*, is a parasitic disease endemic to tropical and subtropical countries of Latin America. Infection is mainly initiated by hematophagous insects that excrete parasite-laden feces near the bite site during feeding [5,6]. The first (acute) phase of the disease manifests in a high level of parasitemia, with the parasites accumulating in peripheral blood and tissue. This phase is frequently asymptomatic or can produce nonspecific symptoms (e.g., nausea, diarrhea, and rashes), making it difficult to diagnose. In the second (chronic) phase, parasites predominantly reside in certain tissues, such as those of the heart, esophagus, colon, and peripheral nervous system. Even though the infection may remain latent for decades, 30% of infected individuals eventually develop cardiac and/or intestinal complications [5,6].

**Figure 1:**
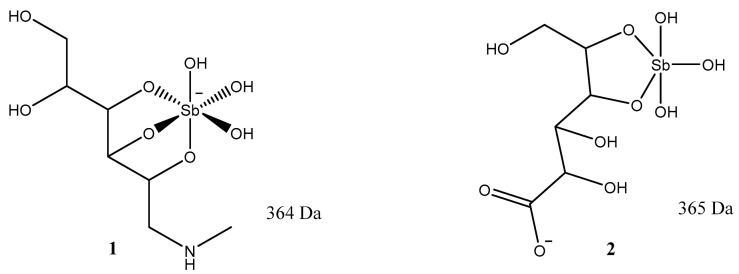
Chemical structures of two antimonials, meglumine antimonate (**1**) and sodium stibogluconate (**2**), utilized for treating leishmaniasis.

The drugs of choice for the treatment of leishmaniasis are antimonials, which are highly toxic [2,3,4]. Due to the amorphous state of antimonials, their chemical structures were unknown until recently. Advances in NMR and mass spectrometry have allowed for the determination of the structures of these compounds. Sb (V) with *N*-methyl-*D*-glucamine is called meglumine antimonate (Glucantime), and Sb (V) with sodium gluconate is called sodium stibogluconate (Pentostam). Recently, the two structures were tentatively identified based on negative electrospray ionization (ESI (-)-MS) [7] (Figure 1).

**Figure 2:**
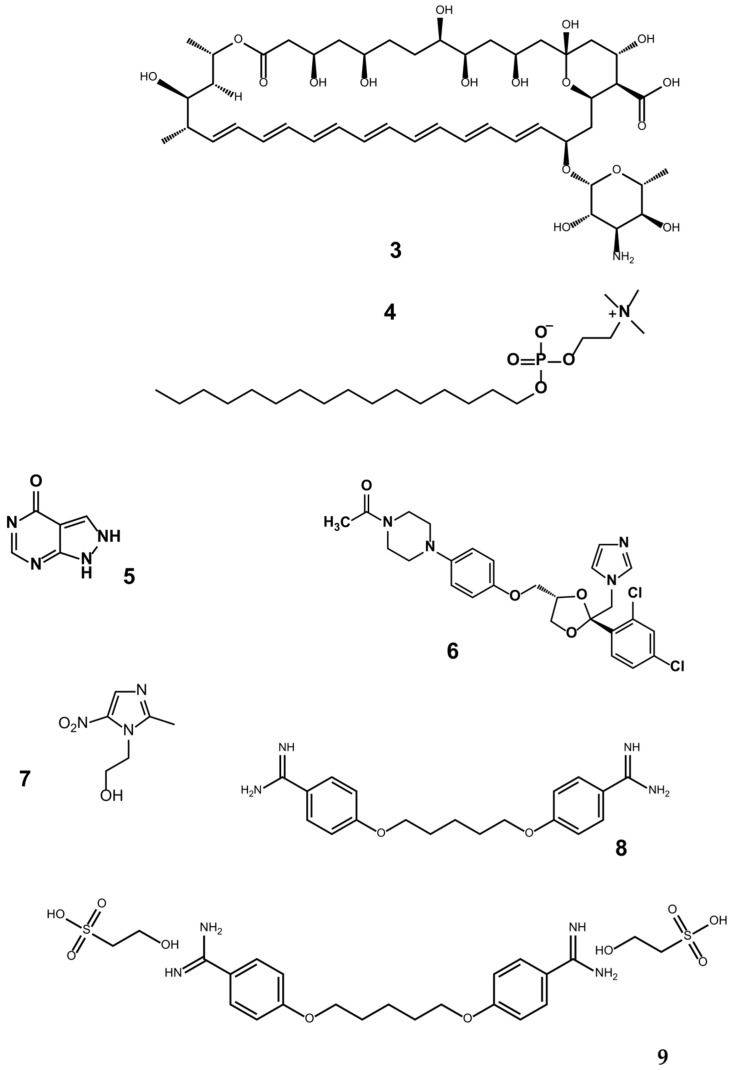
Other drugs tested against *Leishmania*: amphotericin (**3**), miltefosine (**4**), allopurinol (**5**), ketoconazole (**6**), metronidazole (**7**), pentamidine (**8**), and pentamidine isethionate (**9**).

The emerging resistance of *Leishmania* has limited the use of antimonials [4,7], leading to the testing of amphotericin B (**3**), miltefosine (**4**), allopurinol (**5**), ketoconazole (**6**), metronidazole (**7**), pentamidine (**8**), and pentamidine isethionate in different parts of the world [4,8] (Figure 2).

**Figure 3:**
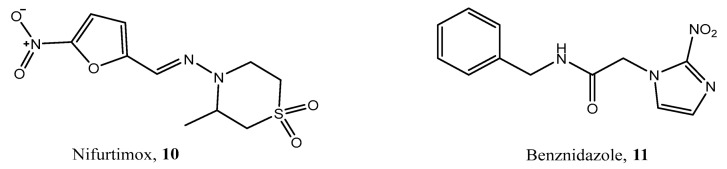
The chemical structures of nifurtimox (**10**) and benznidazole (**11**).

Regarding Chagas disease, the only drugs known to provide successful treatment are nifurtimox (**10**) [9] and benznidazole (**11**) [10] (Figure 3). Unfortunately, these two compounds are expensive and highly toxic [11]. Their multiple adverse effects are often intensified by the long-term administration required for an efficacious treatment. Furthermore, both nifurtimox and benznidazole are only effective at the onset of the disease, not during the chronic phase [12]. Hence, it is important to develop new therapeutic drugs with a low toxicity that are capable of treating the chronic phase as well as the onset of Chagas disease.

Contradictory reports have been published on the toxicity and side effects of pentamidine isethionate (**9**), employed in the treatment of trypanosomiasis and leishmaniasis (Figure 2). Piccica et al. [13] stated that there are very few cases of adverse effects, although fatalities are among them. According to Kuhlmann et al. [14], 50% of patients undergo side effects, which include arrhythmia, syncope, vomiting, hyperglycemia, and reversible kidney damage.

The aim of the current contribution was to synthesize new 2-(4-alkyloxyphenyl)-imidazoles and imidazolines and evaluate them in vitro on *L. mexicana* and *T. cruzi*.

## 2. Results and Discussion

### 2.1. Chemical Basis of Bioisosteres

Since pentamidines have an amidine group in their chemical structure, they are easily modified to promote the formation of nonclassical bioisosteres (imidazolines and imidazoles). The central chain can occupy the lateral position, still as an alkyl group with an aromatic ring. The amidine group closes in imidazolines or imidazoles, thus generating nonclassical bioisosteres of pentamidine. These new compounds could possibly possess better properties than pentamidine. Indeed, the treatment of *T. cruzi* and *L. mexicana* with imidazoles as antiprotozoal compounds has already been reported [15]. Moreover, our group has synthesized some imidazolines and imidazoles and tested them on two bacteria, *Chromobacterium violaceum* and *Serratia marcescens* [16,17,18]. To our knowledge, the compounds herein synthesized have not been examined as either leishmanicidal or trypanocidal agents.

### 2.2. Synthesis of Antiprotozoal Compounds

#### 2.2.1. Synthesis of Imidazolines

The synthesis of imidazolines has been the focus of many studies because numerous compounds with this ring have important applications, ranging from pharmacological therapy to sensors [19,20]. The basis of the present synthesis of imidazolines is the procedure previously described by our group [17,18], improved by adopting MW and ultrasound as energy sources. Firstly (step a), an *n*-alkyl chain was introduced through alkylation of the phenol group of *para*-hydroxy benzaldehyde (Table 1).

With the intermediate on hand, the synthesis of imidazolines from aldehydes is a relatively simple process involving ethylenediamine and an oxidizing agent. When K_2_CO_3_, I_2_, and *t*-BuOH were combined at 70 °C, the reaction took 4–5 h. On the other hand, the reaction of NBS and CH_2_Cl_2_ at room temperature (rt) lasted up to 16 h [21]. These methods afford good to excellent yields, but the reaction times are long. From the chemical point of view, almost no reaction or product is surprising, considering the numerous existing catalysts and novel methods developed to synthesize sophisticated compounds. Given the broad range of options, many researchers in organic synthesis select synthetic routes that employ the principles of green chemistry, with the aim of having the lowest impact on the environment. Accordingly, MW and ultrasound were chosen as energy sources and compared to conventional heating.

As mentioned above, Sant’ Anna et al. [22] described the synthesis of an imidazoline by utilizing ultrasound energy, NBS, and H_2_O. They managed to decrease the reaction time from hours to just minutes. Taking this work as the starting point, some experiments were carried out to synthesize 8-octyloxyphenylimidazoline (Table 2).

The lack of reaction found in experiments B and C could be due to the insolubility of the raw material in water and even in ethanol water. The other outcomes were also unattractive, even though experiment F gave almost the same yield with a drastic reduction in reaction time.

Subsequently, MW and ultrasound energy were used. Imidazolines containing short chains (**22**–**26**) were not included because these compounds provide good yields with both conventional heating and MW. The results of reacting compounds with carbon chains C_5_–C_9_ are shown in Table 3 (**27**–**31**), showing almost the same yields with MW and ultrasound energy, but in half the time with the latter.

#### 2.2.2. Synthesis of Imidazoles

Imidazoles were synthesized by starting from imidazolines and oxidizing the imidazoline ring. Although NBS can work well to oxidize oxazolines to oxazoles, it did not work in this case. The other oxidant utilized was MnO_2_, which has been reported for the synthesis of imidazoles [23,24]. Since the number of equivalents used in the reaction was not specified, the current experiments started with five equivalents, and the concentration was gradually increased to try to improve the yield (Table 4).

Based on the data in Table 4, imidazoles **32**–**36** were synthesized (Table 5).

### 2.3. Synthesis of the Diol

**Figure 4:**
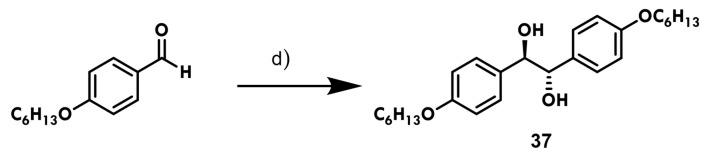
Synthesis of diol **37** d) CrCl_2_, DMF, rt, 120 min and then NH_4_Cl (aq) 5%.

The diol (**37**) was prepared according to our previously reported methodology [25] (Figure 4).

### 2.4. Synthesis of 3-(Bromomethyl)Quinoxaline-2(1H)-One, ***38***

**Figure 5:**
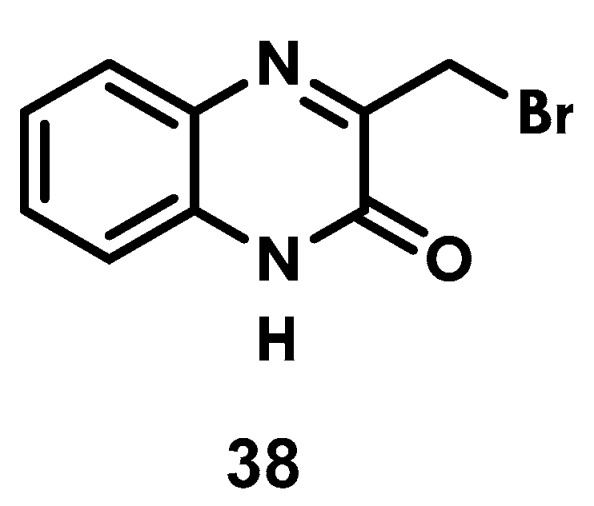
3-(bromomethyl)quinoxaline-2(1*H*)-one (**38**).

3-(bromomethyl)quinoxaline-2(1*H*)-one (**38**) was synthesized with dichloromethane and ethyl 3-bromo-2-oxopropanoate, as usual (Figure 5).

Some compounds containing five-membered heterocycles have been investigated for their leishmanicidal activity. For instance, Fluconazole and Itraconazole are active against leishmaniasis, but the former has serious side effects and the latter showed low effectiveness [4]. Pyrazolo-pyridazinones are active against leishmaniasis when the disease is caused by *Leishmania amazonensis* [26]. In addition, benzimidazole derivatives are selective inhibitors of arginase from Leishmania. The latter derivatives show biological activity against promastigotes and amastigotes [27].

On the other hand, activity against *T. cruzi* has been found for several heterocycle compounds. These include 2-, 4-, and 5-substituted imidazoles with good activity [15] and imidazole-containing nitrophthalazine derivatives [28]. The activity of quinones possessing triazine derivatives is attributed to the reducing power of quinone rather than the heterocycle [29].

### 2.5. Biological Evaluation

#### In Vitro Evaluation of Some Compounds on *Leishmania mexicana* and *Trypanosoma cruzi*

Some of the synthesized compounds (**22**–**23**, **26**–**31**, **33**, **35**–**38**) were chosen to assess their in vitro effect on *L. mexicana* promastigotes and *T. cruzi* epimastigotes, expressed as the half inhibitory concentration (IC_50_). Compounds **37** and **38** were tested only on *T. cruzi*. The cytotoxicity of the compounds was examined on murine macrophages. Based on the resulting data, the selectivity index (SI) was calculated for all thirteen compounds (Table 6). As known, a compound with acceptable activity should have an SI above 10.

The leishmanicidal activity of ten of the thirteen compounds was expressed as an IC_50_ < 10 µg/mL. Among these compounds, **27**, **28**, **29**, **30**, and **31** were the most active, with IC_50_ values < 1 µg/mL (similar to the reference drugs). By relating the leishmanicidal activity to the cytotoxic effect, the SI can be determined. Various researchers suggest that compounds of interest should present an SI ≥ 10 [30], which was the case for all ten compounds. The compound with the greatest leishmanicidal activity was **28**, followed by **30**, **29**, and **31** in descending order.

In the evaluation on epimastigotes of *T. cruzi*, only **30** and **36** reached an IC_50_ < 1 µg/mL, showing better inhibition than both reference drugs. However, compounds **29**, **33**, and **35** also demonstrated attractive trypanocidal activities, with IC_50_ values < 10 µg/mL, similar to the values for Bnz and Nfx. By analyzing the relation of trypanocidal activity to toxicity, a low SI was found for **29**, indicating that it should be discarded. The SI values of **30**, **33**, **35**, and **36** were above 10, with the latter compound being the most selective, surpassing both reference drugs. Compounds **30**, **33**, **35**, and **36** were active against *T. cruzi* and *L. mexicana*. In contrast, **27** and **28** only exhibited leishmanicidal activity.

It is important to measure the cytotoxic effect along with the antiparasitic activity in order to understand the intensity of the effect of compounds on parasite versus mammalian cells (herein represented by a murine macrophage cell line). Mammals constitute the hosts of both parasites. Compounds **22** and **23** exhibited the least cytotoxic effect but are not of interest due to their high IC_50_ values.

### 2.6. Structure–Activity Relationship

When considering the distinct alkyloxy substitution of each imidazoline derivative (**22**–**28** and **30**), the resulting activity on *L. mexicana* promastigotes displayed a clear trend in relation to the molecular structure. Molecules with carbon chains longer than four carbons exhibited IC_50_ values under 1 µg/mL. Compounds **27** (C5), **28** (C6), **29** (C7), and **30** (C8) showed the lowest values, at 0.808, 0.175, 0.2022, and 0.2020 µg/mL, respectively. The trend was similar for *T. cruzi* epimastigotes, although **27** and **28** had a lesser effect. The methyloxy (**22**) and ethyloxy (**23**) derivatives of imidazoline are regarded as inactive because of their elevated IC_50_ values. Compounds with a carbon chain length between four and six carbons (**26**–**28**) afforded double-digit IC_50_ values. The best IC_50_ (0.628 µg/mL) was obtained with the octyloxy derivative (**30**), but **36** also presented a very good IC_50_ (0.6337 µg/mL).

The evaluation of the safety of the compounds on macrophages resulted in significantly higher 50% cellular cytotoxicity concentration (CC_50_) values than the IC_50_ values with respect to *L. mexicana*. Therefore, the SI values were good (>10) in all cases. Given that the IC_50_ values were greater on *T. cruzi*, the corresponding SI values were lower. Of all the imidazolines tested on *T. cruzi*, only **30** (C8) had an SI value > 10, which was better than the 8.31 value found for benznidazole (one of the reference drugs). In contrast, the three imidazoles (**33**, **35**, and **36**) tested on *T. cruzi* gave an SI > 10.

A trend could be observed in the group of hexyl (**33**), octyl (**35**), and nonyloxy (**36**) imidazole derivatives. Longer alkyl chains consistently produced good IC_50_ values against *L. mexicana* and *T. cruzi*. Thus, **36** exhibited the best IC_50_ values for *L. mexicana* (1.095 µg/mL) and *T. cruzi* (0.6337 µg/mL). Another pattern identified was the increased cytotoxicity associated with a greater number of carbons in alkyl substituents. Three imidazoles (**33**–**36**) exhibited a very positive outcome, each with an SI value over 10. The most attractive compound was **36**, with an SI superior to that of each of the two reference drugs.

For the purpose of structural screening, the present study included a diol compound (**37**) containing the previously described hexyloxyphenyl fragment as a dimer, connected through a vicinal alcohol. Taking the inactivity of **37** into account, this fragment was not responsible for the antiprotozoal activity of the other compounds, pointing to the probable importance of the imidazoline and imidazole moieties. Finally, a 3-substituted quinoxalin-2(1*H*)-one derivative (**38**) was examined to determine the selectivity of the method of activity evaluation on *T. cruzi* and *L. mexicana*; as expected, no effect was detected.

## 3. Materials and Methods

### 3.1. General

Reagents and solvents were purchased from Sigma Aldrich (Toluca, Mexico) and used without further purification. The reactions were monitored via thin-layer chromatography (TLC) on Merck F253 silica gel aluminum sheets. Spots were visualized with UV light (254 nm) and iodine. Energy was provided by a Prendo chemical microwave oven MIC-1 (Puebla, Mexico) with a maximum power of 600 W and an ND Scientific ultrasonicator (model A150) with a maximum power of 150 W and frequency of 20–25 KHz. Melting points were determined on an Electrothermal MELT-THEMP apparatus (Electrothermal, Burlington, NJ, USA) and were uncorrected. ^1^H and ^13^C NMR spectra of the compounds were recorded on a Varian NMR System (500 MHz and 125 MHz) and a Varian Mercury (300 MHz and 75 MHz), respectively, assigning the peaks with input from 2D experiments (gHSQC and gHMBC). The chemical shifts (*δ*) are expressed in ppm. MS spectra were acquired on Bruker Amazon Speed apparatus (ESI) (Bruker, Bremen, Germany) via the direct insertion probe–electrospray ionization–mass spectrometry (DIP-ESI-MS) technique. IR spectra were captured on a Perkin Elmer FT-IR Spectrum 2000 spectrometer (Shelton, CT, USA) at the ENCB-IPN spectroscopy instrumentation center. Selected spectra are attached in the Supplementary Materials.

### 3.2. Synthesis of 4-Alkyloxybenzaldehydes

To 30 mL of freshly distilled acetone were added 1 g (8.2 mmol, 1 eq.) of 4-hydroxybenzaldehyde and 2.59 g (16.4 mmol, 2 eq.) of potassium carbonate, and the solution was placed in an ultrasound apparatus (P = 60 W, f = 20–25 KHz) at 40 °C for 30 min. Subsequently, 0.92 mL (9.84 mmol, 1.1 eq.) of haloalkyl was injected and the reaction was continued for 1.5 h under the same conditions. Upon completion of the reaction, the solution was cooled to room temperature. Potassium carbonate was filtered and washed with 150 mL of dichloromethane. The combined organic layers were evaporated under reduced pressure. The crude material was purified with column chromatography on silica gel, using a polarity gradient with different hexane/ethyl acetate ratios (95:5, 9:1, 8:2, and 7:3). The fractions with a pure product were evaporated under reduced pressure and finally dried under vacuum, yielding a yellow liquid that was characterized via ^1^H and ^13^C NMR as well as IR and mass spectrometry. Data from the characterization of the compounds are described hereafter.

**4-Methoxybenzaldehyde (12)**. Yield (93%); mp, oil at rt; IR (KBr) ν = 3070 (C-H aromatic), 2936 (C-H), 1682 (C=O), 1258, 1023 (=C-O-C), 830 (*p*-substituted) cm^−1^; ^1^H NMR (CDCl_3_) *δ* = 9.88 (s, 1H, CHO), 7.41 (AA′BB′, 4H, Ar), 3.88 (s, 3H, OCH_3_); ^13^C NMR (CDCl_3_) *δ* = 191.00 (CHO), 164.73 (C-5), 132.13 (C-3), 130.06 (C-2), 114.43 (C-4), 55.72 (C-6).

**4-Ethyloxybenzaldehyde (13)**. Yield (93%); mp, oil at rt; IR (KBr) ν = 3074 (C-H aromatic), 2983, 2939 (C-H), 1693 (C=O), 1257, 1042 (=C-O-C), 835 (*p*-substituted) cm^−1^; ^1^H NMR (CDCl_3_) *δ* = 9.85 (s, 1H, CHO), 7.80, 6.96 (AA′BB′, 4H, Ar), 4.09 (q, *J* = 7.0 Hz, 2H, OCH_2_), 1.43 (t, *J* = 7.0 Hz, 3H, CH_3_); ^13^C NMR (CDCl_3_) *δ* = 191.0 (CHO), 164.2 (C-5), 132.1 (C-3), 129.8 (C-2), 114.8 (C-4), 64.0 (C-6), 14.8 (C-7).

**4-Propyloxybenzaldehyde (14)**. Yield (95%); mp, oil at rt; IR (KBr) ν = 3074 (C-H aromatic), 2967, 2879 (C-H), 1692 (C=O), 1258, 1044 (=C-O-C), 832 (*p*-substituted) cm^−1^; ^1^H NMR (CDCl_3_) *δ* = 9.80 (s, 1H, CHO), 7.74, 6.91 (AA′BB′, 4H, Ar), 3.92 (t, *J* = 6.6 Hz, 2H, OCH_2_), 1.76 (m, 2H, H-7), 0.98 (t, *J* = 7.7 Hz, 3H, CH_3_). ^13^C NMR (CDCl_3_) *δ* = 190.43 (CHO), 164.0 (C-5), 131.7 (C-3), 129.5 (C-2), 114.5 (C-4), 69.6 (C-6), 22.1 (C-7), 10.1 (C-8).

**4-*iso*-propyl benzaldehyde (15)**. Yield (78%); mp, liquid at rt; IR (KBr) ν = 3073 (C-H aromatic), 2980, 2936 (C-H), 1689 (C=O), 1258, 1007 (=C-O-C), 831 (p-substituted). ^1^H NMR (CDCl3) *δ* = 9.86 (s, 1H, CHO), 7.38 (AA′BB′, 4H, Ar), 4.66 (m, 1H, OCH), 1.37 (d, 6H, CH(CH3)2); ^13^C NMR (CDCl3) *δ* = 190.95 (CHO), 163.29 (C-5), 132.16 (C-3), 129.59 (C-2), 115.68 (C-4), 70.41 (C-6), 21.99 (C-7).

**4-Butyloxybenzaldehyde (16)**. Yield (92%); mp, oil at rt; IR (KBr) ν = 3074 (C-H aromatic), 2958, 2870 (C-H), 1689 (C=O), 1255, 1023 (=C-O-C), 830 (*p*-substituted) cm^−1^; ^1^H NMR (CDCl_3_) *δ* = 9.84 (s, 1H, CHO), 7.79, 6.95 (AA′BB′, 4H, Ar), 4.00 (t, *J* = 6.5 Hz, 2H, OCH_2_), 1.76 (q, 2H, H-7), 1.47 (m, 2H, H-8), 0.95 (t, *J* = 7.4 Hz, 3H, CH_3_); ^13^C NMR (CDCl_3_) *δ* = 190.9 (CHO), 164.3 (C-5), 132.1 (C-3), 129.8 (C-2), 114.8 (C-4), 68.2 (C-6), 31.2 (C-7), 19.3 (C-8), 13.9 (C-9).

**4-Pentyloxybenzaldehyde (17)**. Yield (88%); mp, liquid at rt; IR (film) ν = 3074 (C–H), 2930, 2850 (CHO), 1693 (C=O), 832 (*p*-substituted) cm^−1^. ^1^H NMR (CDCl_3_) *δ* = 9.87 (s, 1H, CHO), 7.82, 6.99 (AA′BB′, 4H, Ar), 4.04 (t, *J* = 6.6 Hz, 2H, H-6), 1.81 (q, 2H, H-7), 1.47 (q, 2H, H-8), 1.35 (m, 4H, H-9 y H-10), 0.91 (t, *J* = 7.1 Hz, 3H, H-11). ^13^C NMR (CDCl_3_) *δ* = 190.9 (C-1), 164.4 (C-5), 132.1 (C-3), 129.9 (C-2), 114.9 (C-4), 68.5 (C-6), 31.6 (C-7), 29.1 (C-8), 25.7 (C-9), 22.7 (C-10), 14.1 (C-11).

**4-Hexyloxybenzaldehyde (18)**. Yield (86%); mp, oil at rt; IR (KBr) ν = 3074 (C-H aromatic), 2932, 2859 (C-H), 1695 (C=O), 1257, 1018 (=C-O-C), 832 (*p*-substituted) cm^−1^. ^1^H NMR (CDCl_3_) *δ* = 9.87 (s, 1H, CHO), 7.82, 6.98 (AA′BB′, 4H, Ar), 4.04 (t, *J* = 6.6 Hz, 2H, OCH_2_), 1.81 (q, 2H, H-7), 1.47 (m, 2H, H-8), 1.34 (m, 4H, H-9; H-10), 0.91 (t, *J* = 7.1 Hz, 3H, CH_3_); ^13^C NMR (CDCl_3_) *δ* = 190.7 (CHO), 164.2 (C-5), 131.9 (C-3), 129.7 (C-2), 114.7 (C-4), 68.4 (C-6), 31.5 (C-7), 29.0 (C-8), 25.6 (C-9), 22.5 (C-10), 13.9 (C-11).

**4-Heptyloxybenzaldehyde (19)**. Yield (80%); mp, oil at rt; IR (KBr) ν = 3074 (C-H aromatic), 2929, 2857 (C-H), 1693 (C=O), 1257, 1016 (=C-O-C), 832 (*p*-substituted) cm^−1^; ^1^H NMR (CDCl_3_) *δ* = 9.85 (s, 1H, CHO), 7.80, 6.97 (AA′BB′, 4H, Ar), 4.01 (t, *J* = 6.6 Hz, 2H, OCH_2_), 1.79 (q, 2H, H-7), 1.44 (m, 2H, H-8), 1.33 (m, 6H, H-9 to H-11), 0.88 (t, *J* = 6.9 Hz, 3H, CH_3_); ^13^C NMR (CDCl_3_); *δ* = 186.1 (CHO), 159.5 (C-5), 127.2 (C-3), 124.9 (C-2), 109.9 (C-4), 68.4 (C-6), 27.0 (C-7), 24.3 (C-8), 24.2 (C-9), 21.1 (C-10), 17.8 (C-11), 9.3 (C-12).

**4-Octyloxybenzaldehyde (20)**. Yield (84%); mp, oil at rt; IR (KBr) ν = 3074 (C-H aromatic), 2925, 2855 (C-H), 1693 (C=O), 1255, 1019 (=C-O-C), 830 (*p*-substituted) cm^−1^; ^1^H NMR (CDCl_3_) *δ* = 9.71 (s, 1H, CHO), 7.66, 6.83 (AA′BB′, 4H, Ar), 3.86 (t, *J* = 6.4 Hz, 2H, OCH_2_), 1.65 (q, 2H, H-7), 1.31 (m, 2H, H-8), 1.18 (m, 8H, H-9 to H-12), 0.76 (t, *J* = 6.9 Hz, 3H, CH_3_); ^13^C NMR (CDCl_3_) *δ* = 189.9 (CHO), 163.7 (C-5), 131.3 (C-3), 129.4 (C-2), 114.2 (C-4), 67.9 (C-6), 31.3 (C-7), 28.9 (C-8), 28.8 (C-9), 28.6 (C-10), 25.5 (C-11), 22.2 (C-12), 13.6 (C-13).

**4-Nonyloxybenzaldehyde (21)**. Yield (81%); mp, oil at rt; IR (KBr) ν = 3074 (C-H aromatic), 2922, 2850 (C-H), 1689 (C=O), 1256, 1033 (=C-O-C), 830 (*p*-substituted) cm^−1^; ^1^H NMR (CDCl_3_) *δ* = 9.84 (s, 1H, CHO), 7.79, 6.95 (AA′BB′, 4H, Ar), 4.00 (t, *J* = 6.6 Hz, 2H, OCH_2_), 1.78 (q, 2H, H-7), 1.43 (m, 2H, H-8), 1.27 (m, 10H, H-9 to H-13), 0.85 (t, J = 6.9 Hz, 3H, CH_3_); ^13^C NMR (CDCl_3_) *δ* = 190.9 (CHO), 164.4 (C-5), 132.1 (C-3), 129.8 (C-2), 114.8 (C-4), 68.5 (C-6), 32.0 (C-7), 29.6 (C-8), 29.5 (C-9), 29.4 (C-10), 29.2 (C-11), 26.1 (C-12), 22.8 (C-13), 14.2 (C-14).

### 3.3. Synthesis of Imidazolines

#### Synthesis of 2-(4-Alkyloxyphenyl)-4,5-Dihydro-1H-Imidazoles

In 20 mL of acetonitrile were dissolved 0.27 g (2 mmol) of 4-alkyloxybenzaldehyde, followed by the addition of 0.16 mL (2.4 mmol, 1.2 eq.) of ethylenediamine. The solution was refluxed at 65 °C for 2 h and monitored via TLC/UV. When no further progress was observed, 0.42 g (2.4 mmol) of NBS was added and the mixture was maintained under reflux for another hour. At the end of the reaction, a 20% KOH solution was added. Subsequently, the product was extracted with ethyl acetate and washed with a 10% NaCl solution. The organic layer was dried over anhydrous Na_2_SO_4_, and the organic phase was evaporated under reduced pressure. The product was purified by crystallization from ethyl acetate and characterized via ^1^H and ^13^C NMR as well as IR and mass spectrometry. Data from the characterization of the compound are described hereafter.

**2-(4-Methoxyphenyl)-4,5-dihydro-1*H*-imidazole (22)**. Yield (80%); mp, 132–134 °C; IR (KBr) ν = 3192 (-NH- aromatic), 2926 (C-H aromatic), 2835 (OMe), 1606 (C-N=C aromatic) cm^−1^; ^1^H NMR (CDCl_3_) *δ* = 7.21 (AA′BB′, 4H, Ar), 5.09 (s, 1H, NH), 3.71 (s, 4H, H-4, H-4′), 3.64 (s, 3H, OCH_3_); ^13^C NMR (CDCl_3_) *δ* = 164.48 (C-2), 161.45 (C-8), 128.71 (C-6, C-6′), 122.37 (C5), 113.61 (C-7, C-7′), 55.25 (C-9), 49.78 (C-4, C-4′); ESI-MS [M + H^+^]: calculated for C_10_H_12_N_2_O: 177.10, measured: 176.90.

**2-(4-Ethoxyphenyl)-4,5-dihydro-1*H*-imidazole (23)**. Yield (82%); mp, 180–182 °C; IR (KBr) ν = 3181 (-NH- aromatic), 2971. 2930 (C-H aromatic), 1619 (C-N=C aromatic), 815 (*p*-substituted) cm^−1^; ^1^H NMR (CDCl_3_) *δ* = 7.30 (AA′BB′, 4H, Ar), 4.02 (c, 2H, H-9), 3.58 (s, 4H, H-4, H-4′), 1.33 (t, 3H, CH_3_); ^13^C NMR (CDCl_3_) *δ* = 168.79 (C-2), 165.34 (C-8), 128.71 (C-6, C-6′), 127.69 (C5), 118.79 (C-7, C-7′), 68.20 (C-9), 54.53 (C-4, C-4′), 19.63 (C-10); ESI-MS [M + H^+^]: calculated for C_11_H_14_N_2_O: 190.11, measured: 190.86.

**2-(4-Propyloxyphenyl)-4,5-dihydro-1*H*-imidazole (24)** Yield (64%); mp, 121–122 °C; IR (KBr) ν = 3189 (-NH- aromatic), 1615 (C-N=C aromatic), 1254 (=C-O-C), 849 (*p*-substituted) cm^−1^; ^1^H NMR (CDCl_3_) *δ* = 7.69, 6.86 (AA′BB′, 4H, Ar), 3.91 (t, 2H, H-9), 3.71 (s, 4H, H-4 y H-4′), 1.79 (m, 2H, H-10), 1.01 (t, *J* = 7.4 Hz, 3H, H-11); ^13^C NMR (CDCl_3_) *δ* = 164.5 (C-2), 161.1 (C-8), 128.5 (C-6), 122.7 (C-5), 114.2 (C-7), 69.2 (C-9), 50.3 (C-4 y C-4´), 22.5 (C-10), 10.6 (C-11); ESI-MS [M + H^+^]: calculated for C_12_H_16_N_2_O: 204.13, measured: 204.89.

**2-(4-*iso*-propyloxyphenyl)-4,5-dihydro-1*H*-imidazole (25)**. Yield (76%); mp, 151–153 °C IR (KBr) ν = 3191 (-NH- aromatic), 2975, 2928 (C-H aromatic), 1615 (C-N=C), 839 (*p*-substituted). ^1^H NMR (DMSO) *δ* = 7.30 (AA′BB′, 4H, Ar), 4.61 (m, 1H, H-9), 3.58 (s, 4H, H-4, H-4′), 1.25 (d, 6H, 2CH_3_). ^13^C NMR (DMSO) *δ* = 163.15 (C-2), 158.88 (C-8), 128.60 (C-6, C-6′), 122.74 (C5), 114.77 (C-7, C-7′), 69.13 (C-9), 40.00 (C-4, C-4′), 21.68 (C-10). MS (DIP-ESI-MS) *m/z* calculated for C_12_H_16_N_2_O (ESI, M + H): 204.13; found: 204.89.

**2-(4-Butyloxyphenyl)-4,5-dihydro-1*H*-imidazole (26)**. Yield (78%); mp, 122–123 °C; IR (KBr) ν = 3197 (-NH- aromatic), 2957, 2921 (C-H aromatic), 1618 (C-N=C aromatic) cm^−1^; ^1^H NMR (CDCl_3_) *δ* = 7.28 (AA′BB′, 4H, Ar), 5.08 (s, 1H, NH), 3.94 (t, 2H, H-9), 3.73 (s, 4H, H-4, H-4′), 1.74 (q, 2H, H-10), 1.46 (m, 2H, H-11), 0.95 (t, 3H, CH_3_); ^13^C NMR (CDCl_3_) *δ* = 164.64 (C-2), 161.36 (C-8), 128.81 (C-6, C-6′), 121.65 (C5), 114.28 (C-7, C-7′), 67.79 (C-9), 49.56 (C-4, C-4′), 31.17 (C-10), 19.19 (C-11), 13.84 (C-12); ESI-MS [M + H^+^] calculated for C_13_H_18_N_2_O: 219.15, measured: 218.97.

**8-Penthyloxyphenil-2-imidazoline (27)**. Yield (80%); mp, 129–131 °C; IR (KBr) ν= 3195 (NH), 2947, 2870 (C–H), 1615 (N–C=N), 1262 (=C–O–C), 846 (*p*-substituted) cm^−1^. ^1^H NMR (DMSO-d_6_) *δ* = 7.74, 6.94 (AA′BB′, 4H, Ar), 3.98 (t, *J* = 6.5 Hz, 2H, H-9), 3.57 (s, 4H, H-4 y H-4′), 1.71 (q, 2H, H-10), 1.36 (m, 4H, H-11 y H-12), 0.89 (t, *J* = 7.1 Hz, 3H, H-13). ^13^C NMR (DMSO-d_6_) *δ* = 163.2 (C-2), 160.2 (C-8), 128.6 (C-6), 122.8 (C-5), 113.9 (C-7), 67.5 (C-9), 49.4 (C-4 y C-4′), 28.3 (C-10), 27.7 (C-11), 21.9 (C-12), 13.9 (C-13). EMBR (DIP-ESI-MS) *m/z* calculated for C_14_H_20_N_2_O (ESI, M + H): 233.16; found: 232.95.

**8-Hexyloxypheny-2-imidazoline (28)**. Yield (71%); mp, 133–135 °C; IR (KBr) ν= 3209 (NH), 2940, 2867 (C–H), 1616 (N–C=N), 1260 (=C–O–C), 849 (*p*-substituted) cm^−1^. ^1^H NMR (CDCl_3_) *δ* = 7.74, 6.85 (AA′BB′, 4H, Ar), 3.93 (t, *J* = 6.6 Hz, 2H, H-9), 3.73 (s, 4H, H-4 y H-4′), 1.74 (q, 2H, H-10), 1.41 (q, 2H, H-11), 1.29 (m, 4H, H-12 y H-13), 0.87 (t, *J* = 7.1 Hz, 3H, H-14). ^13^C NMR (CDCl_3_) *δ* = 164.6 (C-2), 161.4 (C-8), 128.9 (C-6), 121.1 (C-5), 114.2 (C-7), 68.0 (C-9), 49.2 (C-4 y C-4′), 31.4 (C-10), 29.0 (C-11), 25.6 (C-12), 22.5 (C-13), 13.9 (C-14). EMBR (DIP-ESI-MS) *m*/*z* calculated for C_15_H_22_N_2_O (ESI, M + H): 247.18; found: 247.09.

**8-Hepthyloxyphenyl-2-imidazoline (29)**. Yield (64%); mp, 100–102 °C; IR (KBr) ν= 3209 (NH), 2940, 2866 (C–H), 1618 (N–C=N), 1258 (=C–O–C), 848 (*p*-substituted) cm^−1^. ^1^H NMR (CDCl_3_) *δ* = 7.71, 6.86 (AA′BB′, 4H, Ar), 3.94 (t, *J* = 6.5 Hz, 2H, H-9), 3.73 (s, 4H, H-4 y H-4′), 1.76 (q, 2H, H-10), 1.42 (q, 2H, H-11), 1.28 (m, 6H, H-12 al H-14), 0.87 (t, *J* = 6.6 Hz, 3H, H-15). ^13^C NMR (CDCl_3_) *δ* = 164.5 (C-2), 161.2 (C-8), 128.6 (C-6), 122.0 (C-5), 114.2 (C-7), 68.1 (C-9), 49.8 (C-4 y C-4′), 31.7 (C-10), 29.1 (C-11), 29.0 (C-12), 25.9 (C-13), 22.6 (C-14), 14.1 (C-15). EMBR (DIP-ESI-MS) *m*/*z* calculated for C_16_H_24_N_2_O (ESI, M + H): 261.20; found: 261.01.

**8-Octyloxyphenyl-2-imidazoline (30)**. Yield (60%); mp, 102–104 °C; IR (KBr) ν= 3209 (NH), 2924, 2857 (C–H), 1618 (N–C=N), 1246 (=C–O–C), 830 (*p*-substituted) cm^−1^. ^1^H NMR (DMSO-d_6_) *δ* = 7.74, 6.94 (AA′BB′, 4H, Ar), 3.98 (t, *J* = 6.5 Hz, 2H, H-9), 3.57 (s, 4H, H-4 y H-4′), 1.70 (q, 2H, H-10), 1.40 (q, 2H, H-11), 1.25 (m, 8H, H-12 al H-15), 0.85 (t, *J* = 7.0 Hz, 3H, H-16). ^13^C NMR (DMSO-d_6_) *δ* = 163.2 (C-2), 160.2 (C-8), 128.6 (C-6), 122.7 (C-5), 113.9 (C-7), 67.5 (C-9), 49.4 (C-4 y C-4′), 31.2 (C-10), 28.7 (C-11), 28.6 (C-12), 28.6 (C-13), 25.6 (C-14), 22.0 (C-15), 13.9 (C-16). MS (DIP-ESI-MS) *m/z* calculated for C_17_H_26_N_2_O (ESI, M + H): 275.21; found: 275.04.

**8-Nonyloxyphenyl-2-imidazoline (31)**. Yield (62%); mp, 103–105 °C; IR (KBr) ν= 3216 (NH), 2922, 2853 (C–H), 1618 (N–C=N), 1245 (=C–O–C), 828 (*p*-substituted) cm^−1^. ^1^H NMR (CDCl_3_) *δ* = 7.71, 6.89 (AA′BB′, 4H, Ar), 3.96 (t, *J* = 6.6 Hz, 2H, H-9), 3.75 (s, 4H, H-4 y H-4′), 1.77 (q, 2H, H-10), 1.40 (m, 12H, H-11 al H-16), 0.87 (t, *J* = 6.7 Hz, 3H, H-17). ^13^C NMR (CDCl_3_) *δ* = 164.5 (C-2), 161.2 (C-8), 128.6 (C-6), 122.6 (C-5), 114.3 (C-7), 68.2 (C-9), 50.4 (C-4 y C-4′), 31.9 (C-10), 29.6 (C-11), 29.5 (C-12), 29.3 (C-13), 29.2 (C-14), 26.1 (C-15), 22.7 (C-16), 14.2 (C-17). MS (DIP-ESI-MS) *m*/*z* calculated for C_18_H_27_N_2_O (ESI, M + H): 289.23; found: 289.12.

### 3.4. Synthesis of 2-(4-Alkyloxyphenyl)-1H-Imidazoles

**8-Pentyloxyphenyl-2-imidazole (32)**. Yield (67%); mp, 129–131 °C; IR (KBr) ν = 3391 (NH), 2940, 2868 (C–H), 1618 (N–C=N), 1258 (=C–O–C), 844 (*p*-substituted) cm^−1^. ^1^H NMR (CDCl_3_:DMSO-d_6_) *δ* = 7.82, 6.91 (AA′BB′, 4H, Ar), 6.99 (s, 2H, H-4 y H-4′), 3.95 (t, *J* = 6.5 Hz, 2H, H-9), 1.72 (q, 2H, H-10), 1.37 (m, 4H, H-11 al H-12), 0.89 (t, *J* = 7.0 Hz, 3H, H-13). ^13^C NMR (CDCl_3_:DMSO-d_6_) *δ* = 158.5 (C-8), 145.6 (C-2), 126.1 (C-6), 123.3 (C-5), 114.2 (C-7), 113.4 (C-4 y C-4′), 67.3 (C-9), 28.3 (C-10), 27.6 (C-11), 21.8 (C-12), 13.8 (C-13). MS (DIP-ESI-MS) *m*/*z* calculated for C_14_H_18_N_2_O (ESI, M + H): 231.15; found: 231.05.

**8-Hexyloxyphenyl-2-imidazole (33)**. Yield (65%); mp, 133–135 °C; IR (KBr) ν = 3386 (NH), 2952, 2869 (C–H), 1616 (N–C=N), 1253 (=C–O–C), 830 (*p*-substituted) cm^−1^. ^1^H NMR (CDCl_3_:DMSO-d_6_) *δ* = 7.79, 6.83 (AA′BB′, 4H, Ar), 6.96 (s, 2H, H-4 y H-4′), 3.90 (t, *J* = 6.5 Hz, 2H, H-9), 1.70 (q, 2H, H-10), 1.39 (m, 2H, H-11), 1.26 (m, 4H, H-12 y H-13), 0.83 (t, *J* = 6.4 Hz, 3H, H-14). ^13^C NMR (CDCl_3_:DMSO-d_6_) *δ* = 158.3 (C-8), 145.9 (C-2), 125.8 (C-6), 122.8 (C-5), 113.7 (C-7), 113.1 (C-4 y C-4′), 67.1 (C-9), 30.7 (C-10), 28.3 (C-11), 24.8 (C-12), 21.7 (C-13), 13.3 (C-14). MS (DIP-ESI-MS) *m*/*z* calculated for C_15_H_20_N_2_O (ESI, M + H): 245.16; found: 245.08.

**8-Heptyloxyphenyl-2-imidazole (34)**. Yield (57%); mp, 119–121 °C; IR (KBr) ν = 3390 (NH), 2924, 2865 (C–H), 1618 (N–C=N), 1259 (=C–O–C), 840 (*p*-substituted) cm^−1^. ^1^H NMR (CDCl_3_:DMSO-d_6_) *δ* = 7.78, 6.84 (AA′BB′, 4H, Ar), 6.99 (s, 2H, H-4 y H-4′), 3.89 (t, *J* = 6.6 Hz, 2H, H-9), 1.71 (q, 2H, H-10), 1.31 (m, 8H, H-11 al H-14), 0.82 (t, *J* = 6.7 Hz, 3H, H-15). ^13^C NMR (CDCl_3_:DMSO-d_6_) *δ* = 158.3 (C-8), 145.8 (C-2), 125.9 (C-6), 122.5 (C-5), 113.7 (C-7), 113.1 (C-4 y C-4′), 67.1 (C-9), 30.8 (C-10), 28.3 (C-11), 28.1 (C-12), 25.1 (C-13), 21.7 (C-14), 13.3 (C-15). MS (DIP-ESI-MS) *m*/*z* calculated for C_16_H_22_N_2_O (ESI, M + H): 259.18; found: 258.99.

**8-Octyloxyphenyl-2-imidazole (35)**. Yield (51%); mp, 117–119 °C; IR (KBr) ν = 3391 (NH), 2926, 2852 (C–H), 1616 (N–C=N), 1253 (=C–O–C), 830 (*p*-substituted) cm^−1^. ^1^H NMR (CDCl_3_:DMSO-d_6_) *δ* = 7.71, 6.74 (AA′BB′, 4H, Ar), 6.89 (s, 2H, H-4 y H-4′), 3.80 (t, *J* = 6.5 Hz, 2H, H-9), 1.62 (q, 2H, H-10), 1.20 (m, 10H, H-11 al H-15), 0.72 (t, *J* = 6.6 Hz, 3H, H-16). ^13^C NMR (CDCl_3_:DMSO-d_6_) *δ* = 158.9 (C-8), 146.5 (C-2), 126.5 (C-6), 123.1 (C-5), 114.2 (C-7), 113.7 (C-4 y C-4′), 67.7 (C-9), 31.5 (C-10), 29.0 (C-11), 28.9 (C-12), 28.9 (C-13), 25.7 (C-14), 22.3 (C-15), 13.8 (C-16). MS (DIP-ESI-MS) *m*/*z* calculated for C_17_H_24_N_2_O (ESI, M + H): 273.20; found: 273.08.

**8-Nonyloxyphenyl-2-imidazole (36)**. Yield (47%); mp, 110–112 °C; IR (KBr) ν = 3400 (NH), 2925, 2854 (C–H), 1618 (N–C=N), 1258 (=C–O–C), 844 (*p*-substituted) cm^−1^. ^1^H NMR (CDCl_3_) *δ* = 7.77, 6.90 (AA′BB′, 4H, Ar), 7.09 (s, 2H, H-4 y H-4′), 3.95 (t, *J* = 6.6 Hz, 2H, H-9), 1.77 (q, 2H, H-10), 1.44 (m, 2H, H-11), 1.28 (m, 10H, H-12 al H-16), 0.88 (t, *J* = 6.7 Hz, 3H, H-17). ^13^C NMR (CDCl_3_) *δ* = 159.5 (C-8), 146.9 (C-2), 126.6 (C-6), 122.8 (C-5), 114.7 (C-7), 114.2 (C-4 y C-4′), 68.1 (C-9), 31.8 (C-10), 29.5 (C-11), 29.4 (C-12), 29.2 (C-13), 29.2 (C-14), 26.0 (C-15), 22.6 (C-16), 14.1 (C-17). MS (DIP-ESI-MS) *m/z* calculated for C_17_H_24_N_2_O (ESI, M + H): 273.20; found: 273.09.

### 3.5. Synthesis of (1R,2S)-1,2-Bis(4-Alkyloxyphenyl)Ethane-1,2-Diol (***37***)

Into 10.5 mL of dry DMF in a 50 mL two-neck flask under a nitrogen atmosphere, 0.58 g (4.74 mmol, 1 eq) of CrCl_2_ was dissolved. Subsequently, 0.65 mL (4.74 mmol, 2 eq) of dry ethylenediamine in 1.75 mL of dry DMF was added slowly and dropwise into the reaction mixture over 30 min, allowing for the in situ formation of [Cr(en)_2_] ^2+^ at room temperature. After adding the entire amount of DMF, 0.53 mL (4.74 mmol, 0.48 eq) of 4-decyloxybenzaldehyde and 1.74 mL of dry DMF were added and the solution was stirred for another 4 h. The progression of the reaction was monitored via TLC, using a mixture of hexane and ethyl acetate (8:2). Spots were visualized with ammonium molybdate. Upon completion of the reaction, it was quenched with 65.67 mL of a saturated aqueous NH_4_Cl solution. The mixture was stirred for 16 h and left to rest for 15 min. The solid and liquid phases were separated, and the solid phase was extracted with 200 mL of acetone and dried with anhydrous sodium sulfate (Na_2_SO_4_). The solvent was filtered and removed under reduced pressure. The resulting yellow brown amorphous solid was recrystallized from a mixture of ethyl acetate and hexane to afford a pale yellow solid.

**(1*R*,2*S*)-1,2-bis(4-hexyloxyphenyl)ethane-1,2-diol (37)**. Yield (51%); mp, 122–123 °C; IR (KBr) ν = 3338 (OH), 2955, 2854 (C-H), 1257, 1035 (=C-O-C), 1117 (s-OH), 830 (*p*-substituted) cm^−1^; ^1^H NMR (DMSO-d_6_:CDCl_3_ 3:1) *δ* = 7.08, 6.71 (AA′BB′, 8H, Ar), 4.51 (s, 2H, H-1; H-2), 3.88 (t, *J* = 6.5 Hz, 4H, H-7), 1.69 (q, 4H, H-8), 1.31 (m, 12H, H-9 to H-11), 0.87 (t, *J* = 7.0 Hz, 6H, H-12); ^13^C NMR (DMSO-d_6_:CDCl_3_ 3:1) *δ* = 158.4 (C-6), 135.5 (C-3), 129.0 (C-4), 113.9 (C-5), 77.5 (C-1; C-2), 68.1 (C-7), 31.9 (C-8), 29.6 (C-9), 26.1 (C-10), 22.9 (C-11), 14.6 (C-12). HRMS: calculated for C_26_H_38_O_4_: 390.2796, measured: 390.2759.

### 3.6. Synthesis of 3-(Bromomethyl)Quinoxalin-2(1H)-One (***38***) 

Into 20 mL of dichloromethane were dissolved 0.3 g (2.7 mmol) 1,2-phenylenediamine and 0.41 mL (3.3 mmol, 1.2 eq) bromoethyl pyruvate, followed by stirring for 2 h. The crude product that precipitated was collected by filtration and dried under reduced pressure. The pure product was obtained through recrystallization from dichloromethane and characterized via ^1^H and ^13^C NMR as well as IR and mass spectrometry. Data from the characterization of the compound are described hereafter.

**3-(Bromomethyl)quinoxalin-2(1*H*)-one (38)**. Yield (86%); mp, 224–226 °C; IR (KBr) ν = 3919 (N-H), 1662 (C=O), 1609 (C-Br), 763 (*o*-substitution) cm^−1^; ^1^H NMR (DMSO-d_6_:CDCl_3_ 3:1) *δ* = 7.73 (d, 1H, H-8), 7.54 (t, 1H, H-7), 7.30 (m, 2H, H-6 y H-5), 4.59 (s, 2H, H-4); ^13^C NMR (DMSO-d_6_:CDCl_3_ 3:1) *δ* = 156.4 (C-3), 153.6 (C-2), 132.7 (C-5), 131.5 (C-10), 131.1 (C-7), 128.7 (C-6), 123.7 (C-8), 115.6 (C-9), 29.8 (C-4); ESI-MS [M + Na^+^]: calculated for C_9_H_7_BrO: 260.96, measured: 260.88.

### 3.7. Biological Evaluation

#### 3.7.1. Dilution of the Compounds

All compounds were dissolved in dimethylsulfoxide (DMSO) to reach a concentration of 10 mg/mL. From the stock solution, dilutions in phosphate-buffered saline (PBS) were prepared at the time of the biological testing. DMSO did not exceed a concentration of 1% in any of the assays on the biological activity of the compounds. This level is considered to be within the range appropriate for avoiding toxicity in a vertebrate host [30].

#### 3.7.2. In Vitro Evaluation of the Metabolic Inhibition of *Leishmania mexicana* by Fluorometric Analysis with Resazurin

A promastigote culture of *L. mexicana* was harvested in the stationary phase after 7 days of growth in culture. To each well of a sterile 96-well microplate, 5 × 10^5^ promastigotes and the compound to be assessed (or the reference drug) were added to a final volume of 100 μL in RPMI culture medium supplemented with 10% SFB and 1% ampicillin/streptomycin. Each compound was examined at different concentrations, obtained by serial two-fold dilutions starting from 50 μg/mL. The microplate was incubated in the dark at 27 °C for 24 h. Promastigotes with 0.5% DMSO (at the vehicle concentration of the greatest dilution of the compound) served as the positive viability control and amphotericin B at a final concentration of 5 μg/mL served as the negative viability control. Each condition was assayed in triplicate.

After 24 h of incubation, 10 μL of 0.01% resazurin was added to each of the wells and incubation continued for another 24 h under the same conditions. The plate was read via a fluorometer (Spectramax M3, Molecular Devices, LLC, San Jose, CA, USA) at 544 nm excitation and 590 nm emission. Each treated well was compared to the positive viability control, and metabolic inhibition was expressed as a percentage. The IC_50_ value was determined with the Probit statistical tool version 4.1 [31,32,33].

Resazurin is a blue, permeable, and non-fluorescent compound. Upon entering cells, it is reduced to resorufin due to the reducing environment of the cytosol in living cells. Resorufin is red and highly fluorescent. The number of living cells is proportional to the fluorescence intensity of resorufin [31].

#### 3.7.3. In Vitro Evaluation of the Metabolic Inhibition of *Trypanosoma cruzi* INC-5 by Colorimetric Analysis with 3-(4,5-Dimethyl-2-Thiazoyl)-2,5-Diphenyltetrazolic Bromide (MTT)

Epimastigotes of *T. cruzi* INC-5 were harvested after 7 days of growth in culture. In each well of a sterile 96-well microplate, 1 × 10^6^ epimastigotes and the compound to be tested (or the reference drug) were added to a final volume of 100 μL in brain heart infusion (BHI) culture medium supplemented with 10% FBS and 1% ampicillin/streptomycin. Each compound was examined at various concentrations prepared through serial two-fold dilutions starting from 50 μg/mL. The microplate was incubated in the dark at 27 °C for 24 h. Epimastigotes with 0.5% DMSO served as the positive viability control and the reference drugs nifurtimox (Lampit, Bayer, Germany) and benznidazole (Rochagan, Roche, Brazil) served as the negative viability control. Each condition was assayed in triplicate.

Upon completion of the incubation time, 10 μL of MTT solution at 5 mg/mL was added to each well, and incubation continued in the dark at 27 °C for 20 h. Then, 100 μL of a solution containing 10% SDS and 0.01 M HCl was added to stop the reaction. To dissolve the formazan crystals formed by the metabolism of viable epimastigotes, the mixture was left to stand for 4 h. The plates were read on a spectrophotometer (Spectramax Plus, Molecular Devices) at an absorbance of 570 nm. The result found in each well with a given concentration of the respective compound was compared to the positive viability control and the metabolic inhibition was expressed as a percentage. The IC_50_ was determined using the Probit statistical tool [33].

MTT is a yellow, water-soluble compound. In living cells, i8 is captured and reduced to its insoluble form (formazan, which is purple) by mitochondrial succinate dehydrogenase. The quantity of living cells is proportional to the amount of formazan produced [34].

#### 3.7.4. In Vitro Evaluation of the Cytotoxic Effect of the Compounds on Macrophages, Measured by Fluorometric and Colorimetric Analysis with Resazurin

In each well of a 96-well plate, 5 × 10^4^ J774A.1 macrophages were added to a final volume of 100 μL in RPMI culture medium supplemented with 10% FBS, 1% ampicillin/streptomycin, and 1% MEM-NEAA. The microplate was incubated at 37 °C with 5% CO_2_ and humidity for 24 h to allow for the formation of a cell monolayer with a confluence of 80%. Subsequently, a concentration–response evaluation was carried out. Briefly, one of the compounds at the corresponding concentration was placed in each well with macrophages. Each compound was examined at concentrations starting from 100 μg/mL. Each concentration, assayed in triplicate, was prepared by serial dilutions until reaching 50 μg/mL. As the negative cytotoxicity control, cells were exposed to 1% DMSO. The microplate was incubated at 37 °C with 5% CO_2_ and humidity for 20 h. Afterwards, 10 μL of 0.01% resazurin was added and the microplate was incubated for 4 h. As mentioned above, the microplate was read via a fluorometer at 544 nm excitation and 590 nm emission. Each treated well was compared to the negative cytotoxicity control and the data were expressed as a percentage of metabolic inhibition. The Probit statistical tool was used to calculate the CC_50_.

#### 3.7.5. Determination of the Selectivity Index (SI)

It is advantageous to determine the SI as an indicator of how many times more toxic a compound is to the parasite than to the host cell. The SI is the ratio of the CC_50_ for mammalian cells to the IC_50_ for the parasite (CC_50_/IC_50_). In general, the biological activity is considered as good with an SI ≥ 10 [32].

## 4. Conclusions

Two new and potent antiprotozoal compounds were presently identified by synthesizing and evaluating a set of 2-(4-alkyloxyphenyl)-imidazoles and imidazolines. Several compounds exhibited in vitro activity against *L. mexicana* and *T. cruzi*. The best results were achieved with 2-(4-pentyloxyphenyl)-4,5-dihydro-1*H*-imidazole (**17**) against *L. mexicana* promastigotes and 2-(4-octyloxyphenyl)-4,5-dihydro-1*H*-imidazole (**20**) against *T. cruzi* epimastigotes, with SI values of 166 for *T. cruzi* and 15 for *L. mexicana*. Since nifurtimox and benznidazole are the only effective compounds for the treatment of Chagas disease, compound **17** merits further investigation in vivo. The effect on macrophages represents an initial exploration of the safety of the compound. Other cytotoxicity tests will need to follow, including in vivo toxicological studies, specifically an escalating dose experiment. Hence, the current study to find new antiprotozoals constitutes an initial study in which a promising new candidate for the treatment of two neglected tropical diseases was found.

## Figures and Tables

**Table 1:** Results of the alkylation of *p*-hydroxybenzaldehyde (**12**–**21**).

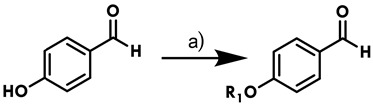	
**Compound**	**Yield (%)**
**12**: R_1_ = CH_3_	95
**13**: R_1_ = C_2_H_5_	95
**14**: R_1_ = C_3_H_7_	92
**15**: R_1_ = * i*C_3_H_7_	90
**16**: R_1_ = C_4_H_9_	90
**17**: R_1_ = C_5_H_11_	89
**18**: R_1_ = C_6_H_13_	88
**19**: R_1_ = C_7_H_15_	86
**20**: R_1_ = C_8_H_17_	86
**21**: R_1_ = C_9_H_19_	85

a) K_2_CO_3_, R_1_-Br, acetone, microwave 60 W, 40 °C, 30 min.

**Table 2:** Comparison of the results of the synthesis of 8-octyloxyphenylimidazoline (**30**) with conventional heating and microwave energy.

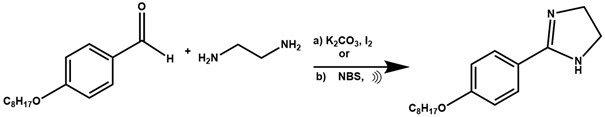				
**Experiment**	**Reagents and Reaction Conditions**	**Solvent**	**Time**	**Yield (%)**
**A**	a*	*t*-BuOH	4 h	61
**B**	b	H_2_O	2 h	NR
**C**	b	H_2_O:EtOH (1:1)	2 h	NR
**D**	b	CH_2_Cl_2_	24 min	34
**E**	b	AcOEt	24 min	48
**F**	b	CH_3_CN	20 min	60

a*, conventional heating at 70 °C; b, 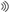: Ultrasound (P = 60 W, f = 20–25 KHz); NR, no reaction and the recovery of the raw materials.

**Table 3:** Results of reactions to obtain 8-alkyloxyphenyl-2-imidazolines (**27**–**31**) with MW and ultrasound energy.

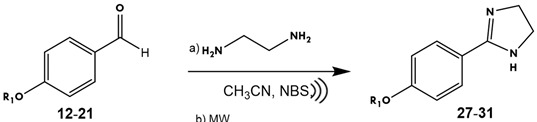					
**Experiment**	**R**	**Energy**	**T (°C)**	**t (min)**	**Yield (%)**
**A**	**27:** C_5_H_11_	MW	50	40	80
**B**	**28:** C_6_H_13_				70
**C**	**29:** C_7_H_15_				62
**D**	**30:** C_8_H_17_				61
**E**	**31:** C_9_H_19_				61
**F**	**27:** C_5_H_11_	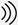	50	20	80
**G**	**28:** C_6_H_13_				71
**H**	**29:** C_7_H_15_				64
**I**	**30:** C_8_H_17_				60
**J**	**31:** C_9_H_19_				62

MW: Microwave energy (P = 360 W), 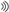: Ultrasound (P = 60 W, f = 20–25 KHz).

**Table 4:** Results of distinct forms of oxidation employed to obtain 8-hepthyloxyphenyl-2-imidazole (**34**).

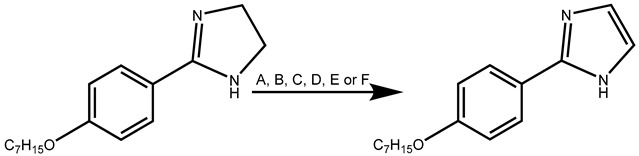					
**Experiment**	**Reagents**	**Energy**	**T (°C)**	**t (min)**	**Yield %**
**A**	NBS, K_2_CO_3_, CH_2_Cl_2_	Δ	Reflux	60	NR
**B**	MnO_2_ (5 eq), toluene	Δ	65	960	NR
**C**	MnO_2_ (5 eq), CH_2_Cl_2_	Δ	RT	960	NR
**D**	MnO_2_ (5 eq), toluene	MW	65	110	10
**E**	MnO_2_ (12 eq), toluene	MW	65	110	22
**F**	MnO_2_ (18 eq), toluene	MW	65	80	57

Δ, conventional heating; MW, microwave energy; NR, no reaction observed.

**Table 5:** Results of the synthesis of 8-alkyloxyphenyl-2-imidazoles (**32**–**36**).

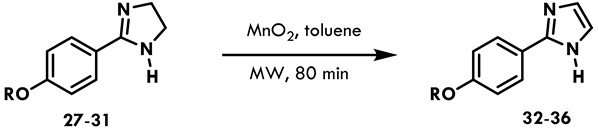		
**Experiment**	**R**	**Yield (%)**
**A**	**32:** C_5_H_11_	67
**B**	**33:** C_6_H_13_	65
**C**	**34:** C_7_H_15_	57
**D**	**35:** C_8_H_17_	51
**E**	**36:** C_9_H_19_	47

MW (P = 360 W).

**Table 6:** Effect of the synthesized compounds on *L. mexicana* promastigotes, *T. cruzi* epimastigotes, and murine macrophages.

	*L. mexicana* Promastigotes	*T. cruzi* INC-5 Epimastigotes	Macrophages J774	SI (CC_50_/IC_50_)	
	IC_50_ (µg/mL)	IC_50_ (µg/mL)	CC_50_ (µg/mL)	*L. mexicana*	*T. cruzi*
**(22)**	16.04 (14.65–17.42)	>50	>100	>6.23	ND
**(23)**	7.62 (6.01–9.22)	>50	>100	>13.12	ND
**(26)**	2.63 (2.39–2.87)	48.74 (45.88–51.61)	>100	>37.98	>0.48
**(27)**	0.808 (0.747–0.868)	37.52 (33.90–41.15)	71.33 (68.33–74.33)	88.28	1.90
**(28)**	0.175 (0.098–0.253)	25.09 (21.098–29.083)	29.21 (25.94–32.48)	166.31	1.16
**(29)**	0.2022 (0.125–0.279)	4.08 (2.15–6.02)	21.48 (18.37–24.59)	106.21	5.25
**(30)**	0.2020 (0.183–0.2207)	0.6284 (0.580–0.660)	9.72 (8.99–10.45)	48.11	15.46
**(31)**	0.5468 (0.4840–0.6096)	21.03 (19.64–22.42)	50.61 (47.63–53.60)	92.56	2.40
**(33)**	3.40 (3.115–3.64)	6.86 (5.36–8.36)	85.74 (80.95–90.53)	25.21	12.58
**(35)**	2.694 (2.47–2.91)	2.109 (1.959–2.259)	40.18 (37.58–42.79)	14.92	19.05
**(36)**	1.095 (1.038–1.153)	0.6337 (0.2137–1.053)	31.28 (28.28–34.27)	28.53	49.35
**(37)**	>50	>50	ND	ND	ND
**(38)**	>50	>50	ND	ND	ND
AmB	0.19 (0.16–0.21)	-	48.12 (46.89–49.35)	253.26	-
Bnz	-	11.02 (9.52–12.52)	91.61 (87.18–96.04)	-	8.31
Nfx	-	2.50 (1.4–3.6)	57.76 (54.18–61.34)	-	23.10

The values in parentheses represent the confidence interval determined by the Probit method with 96% confidence; ND: not determined; AmB = amphotericin; Bnz = benznidazole; Nfx = nifurtimox. All tests were performed in triplicate.

## Data Availability

Data is contained within the article and Supplementary Materials.

## Supplementary Materials

The following supporting information can be downloaded at: https://www.mdpi.com/article/10.3390/ijms25073673/s1.
