# Supplementary figures and images for: Synthesis of Antiprotozoal 2-(4-Alkyloxyphenyl)-Imidazolines and Imidazoles and Their Evaluation on Leishmania mexicana and Trypanosoma cruzi
Source: Int J Mol Sci. 2024 Mar 26;25(7):3673. doi: 10.3390/ijms25073673 (PMC11012064; doi:10.3390/ijms25073673)

## Supplementary material

### Selected Spectra

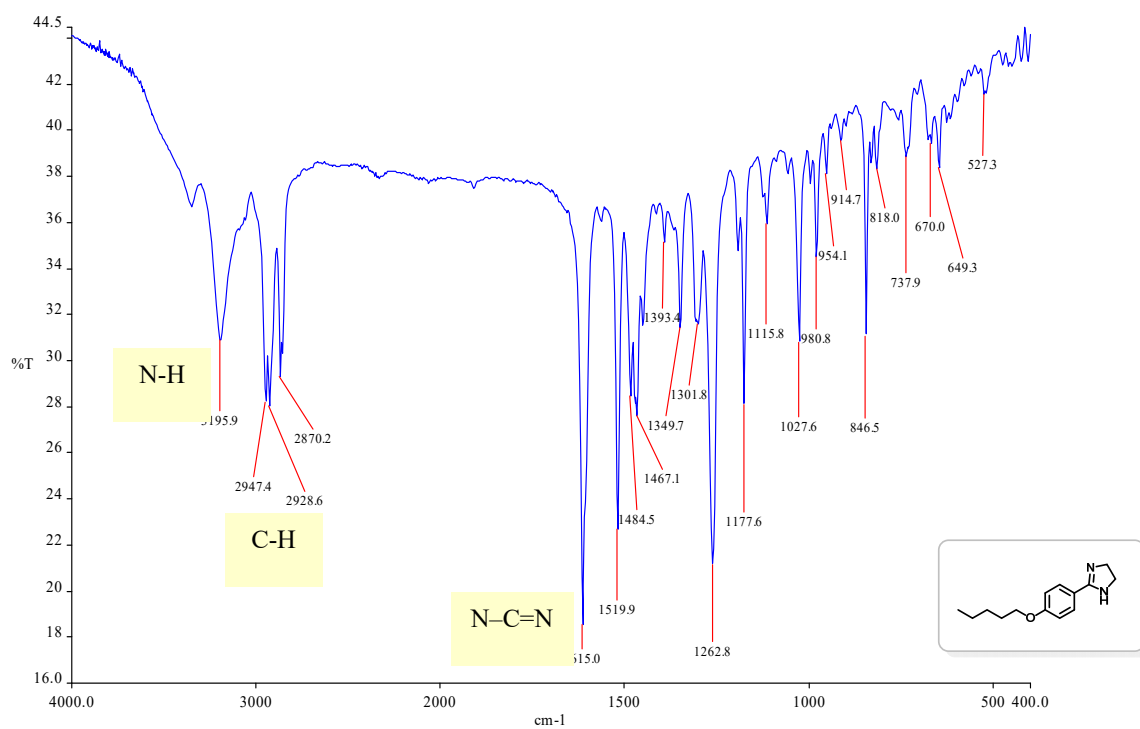

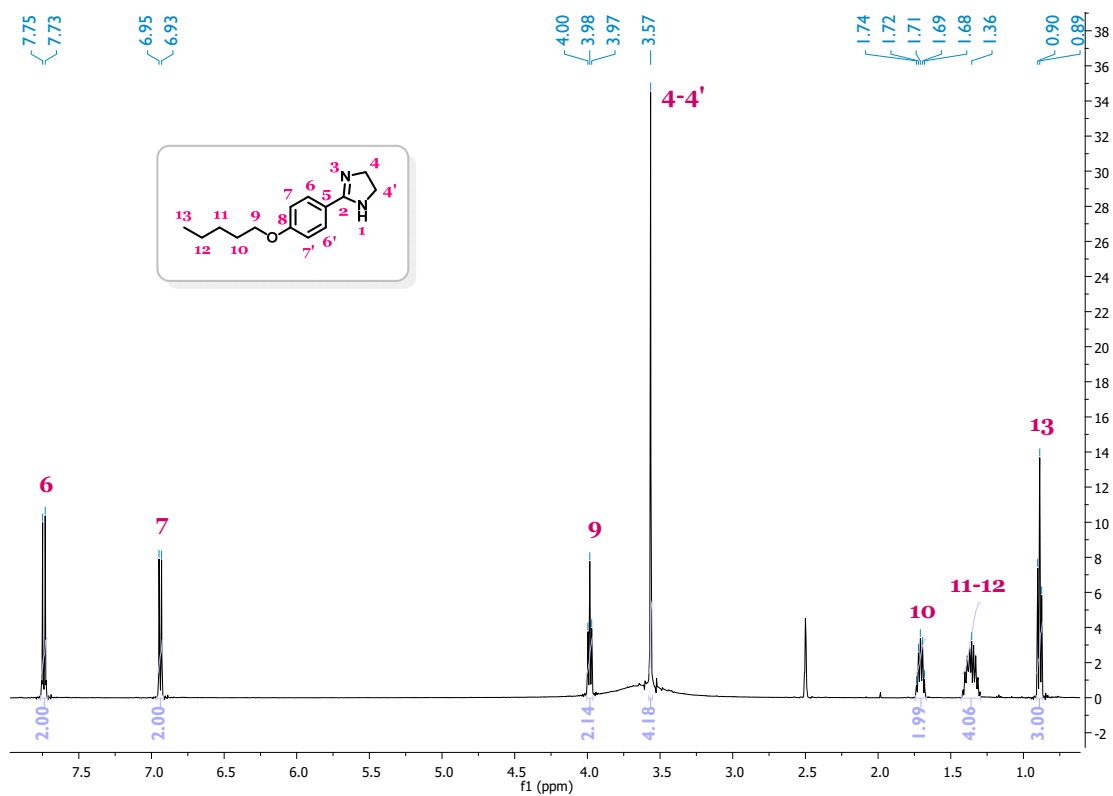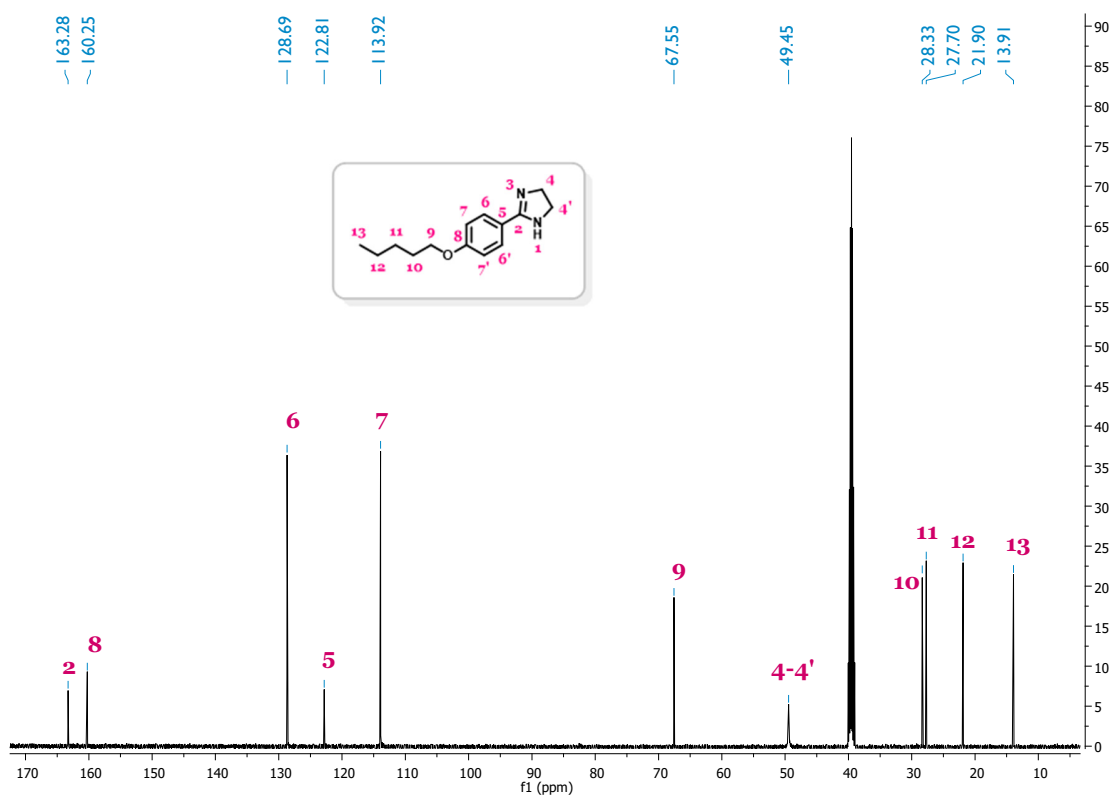

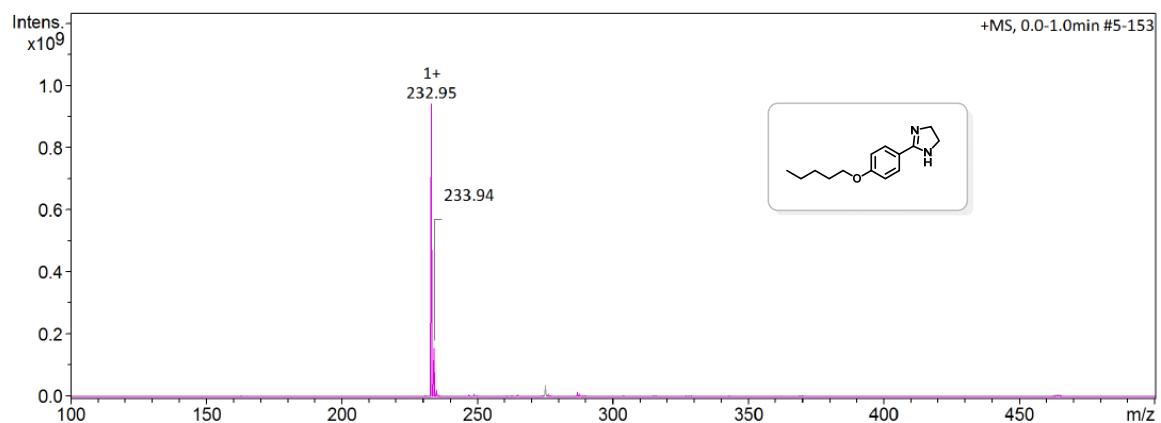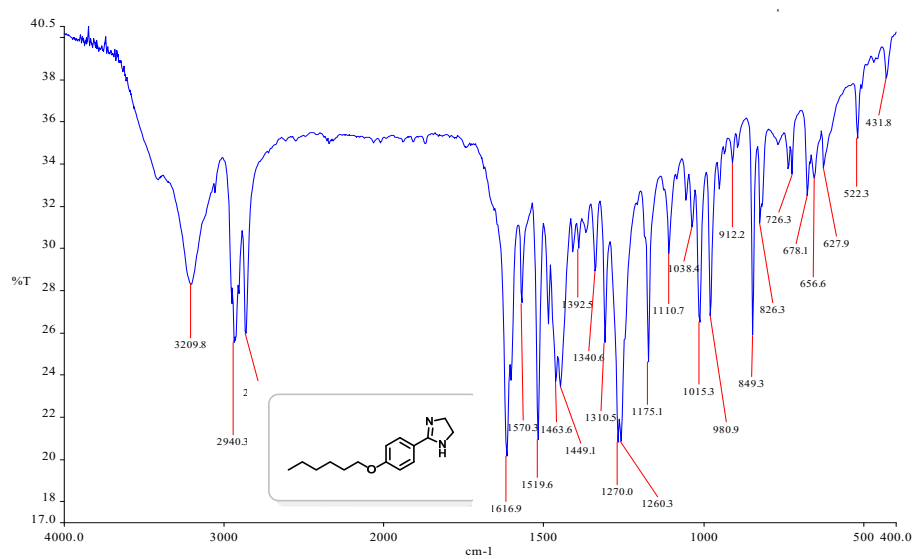

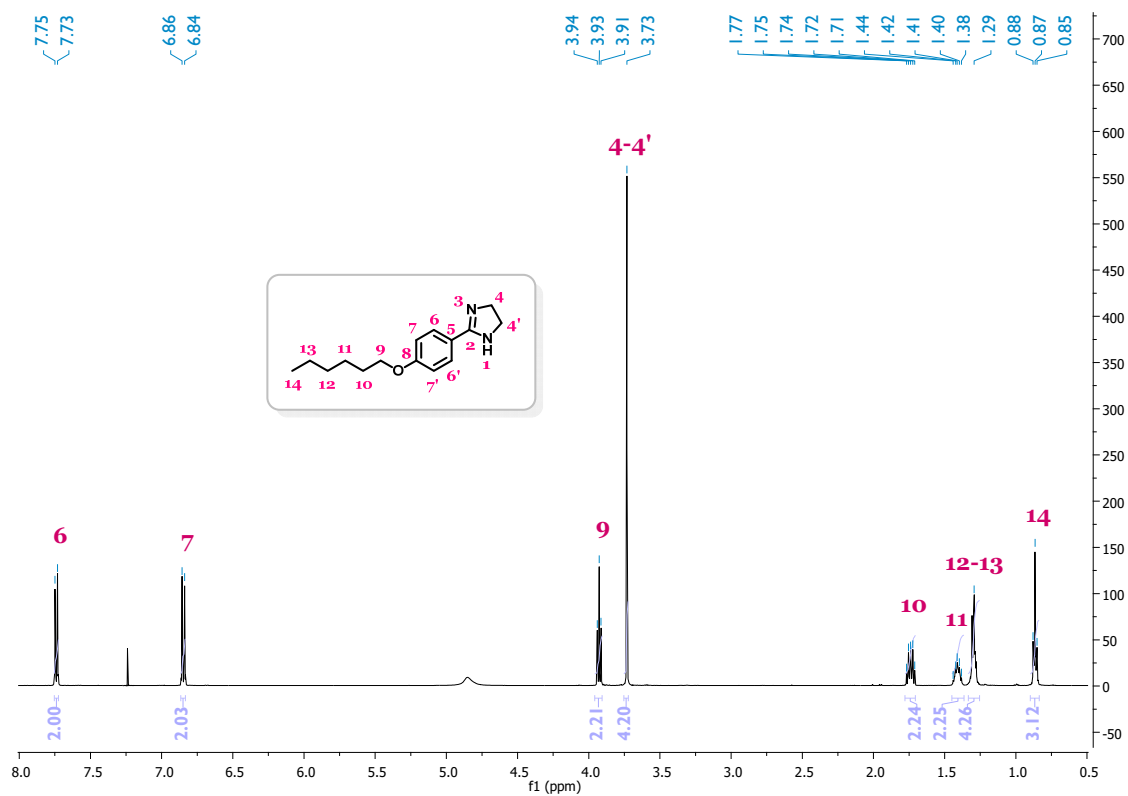

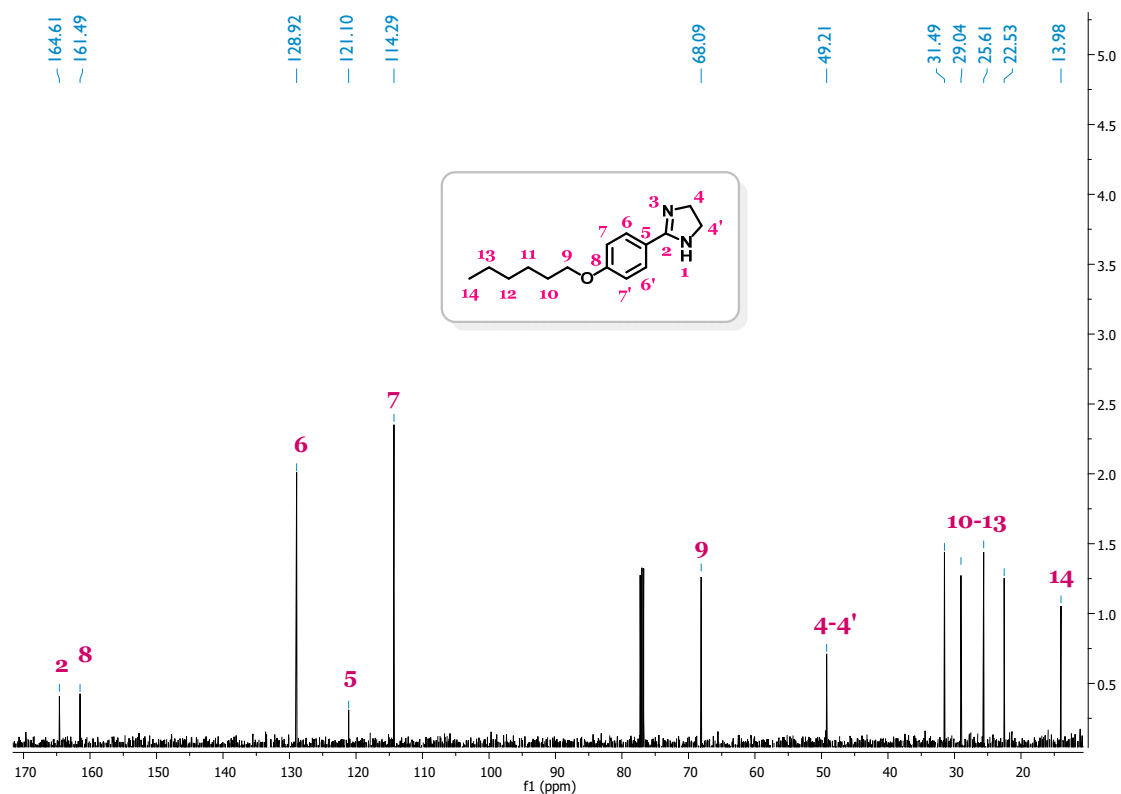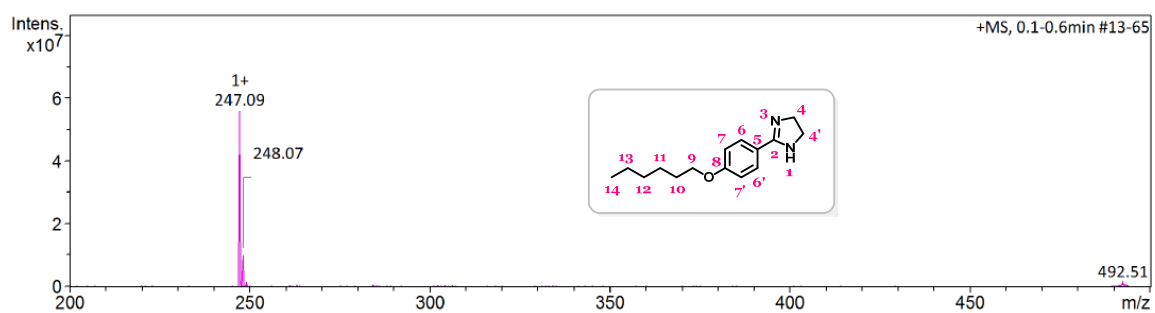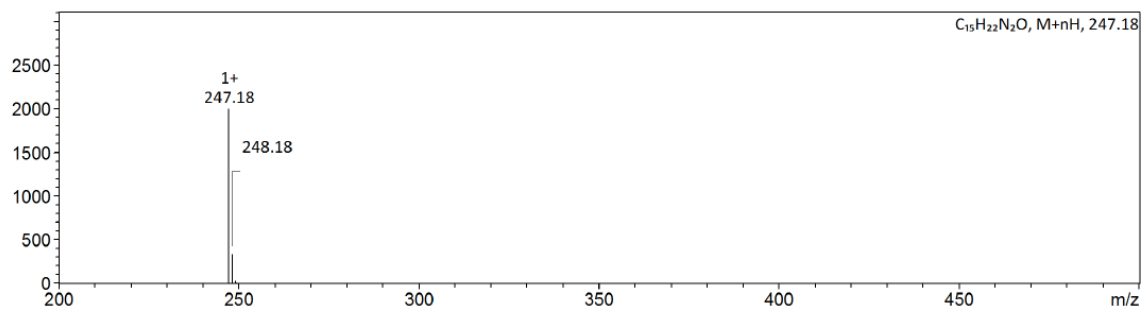

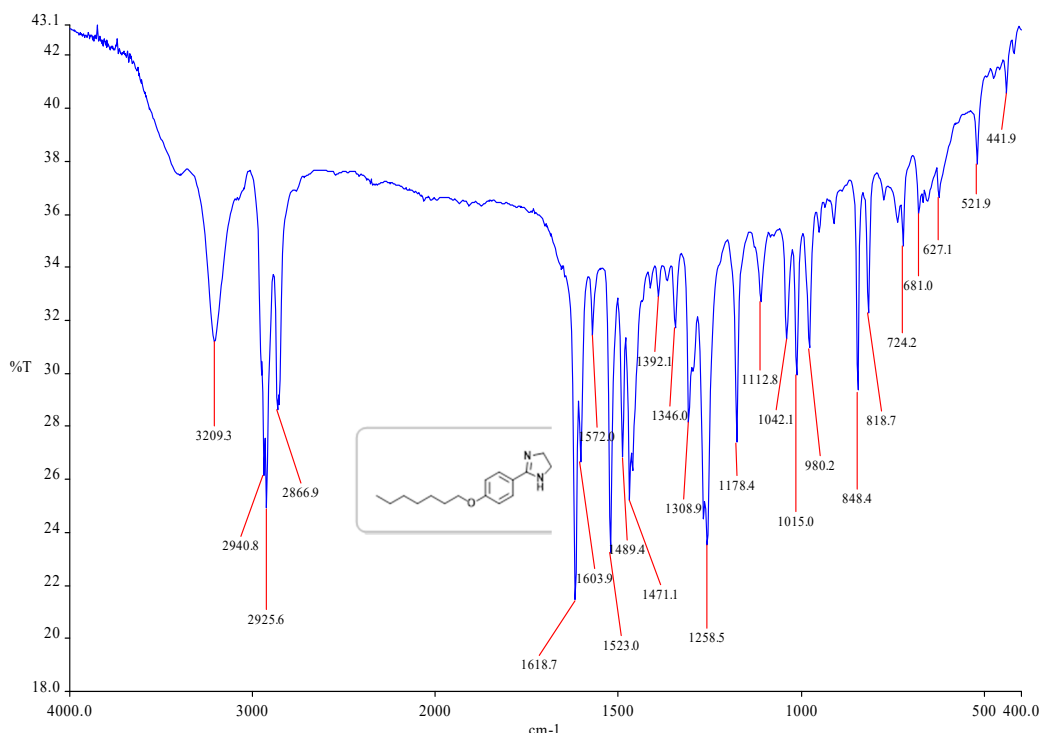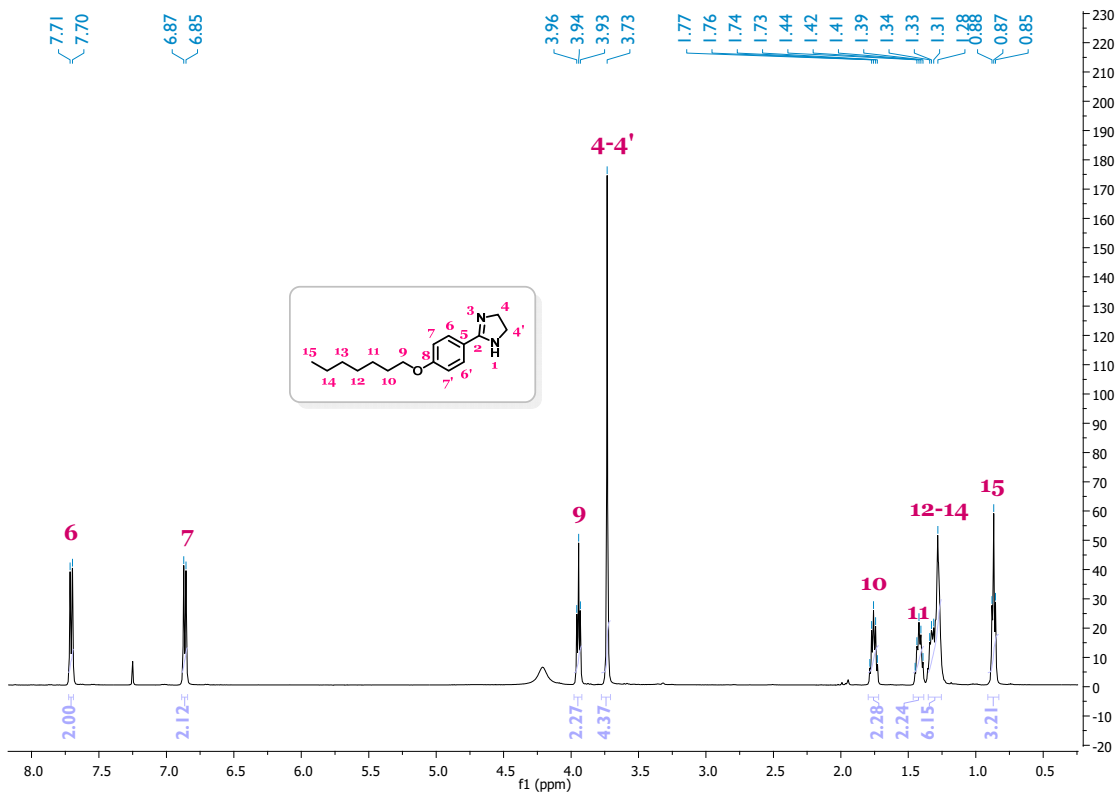

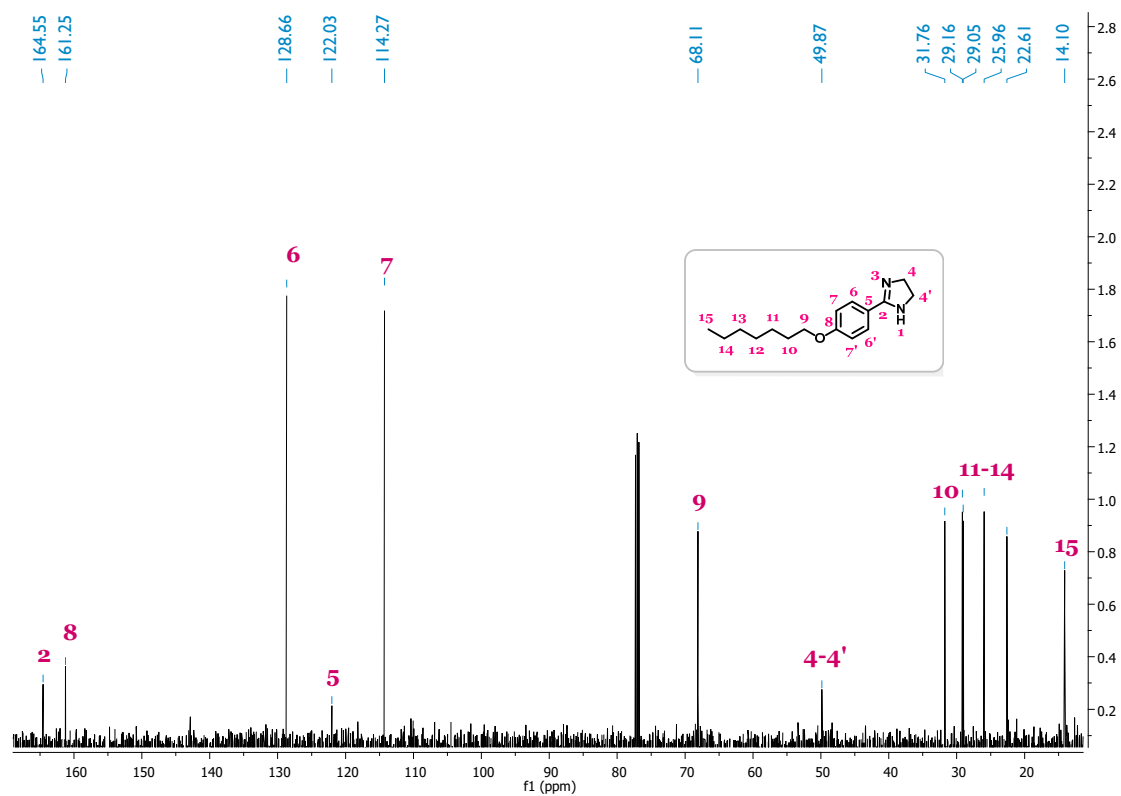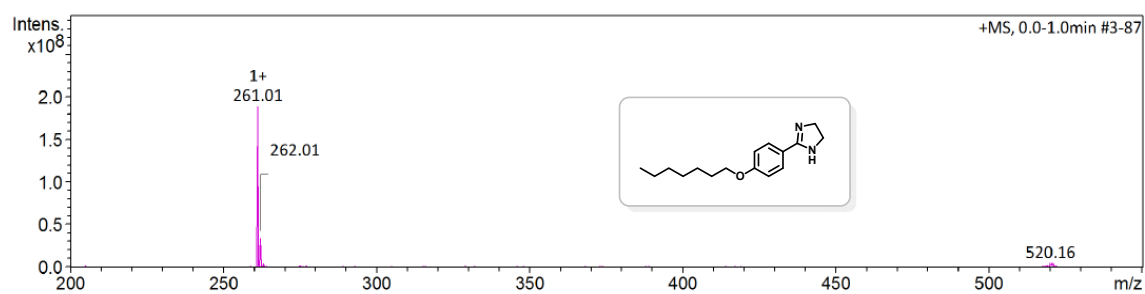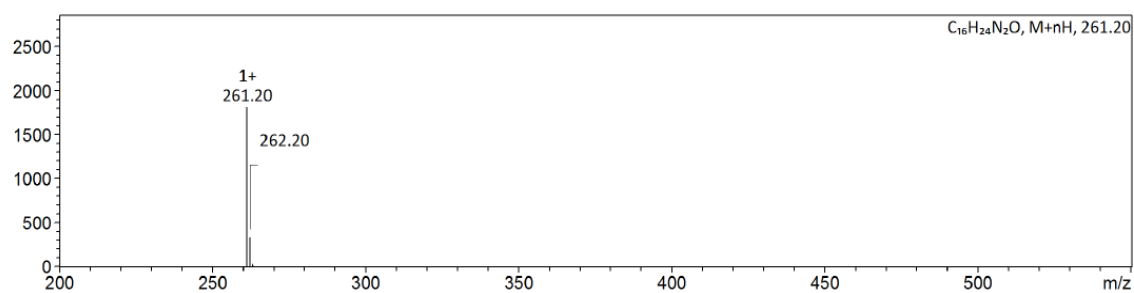

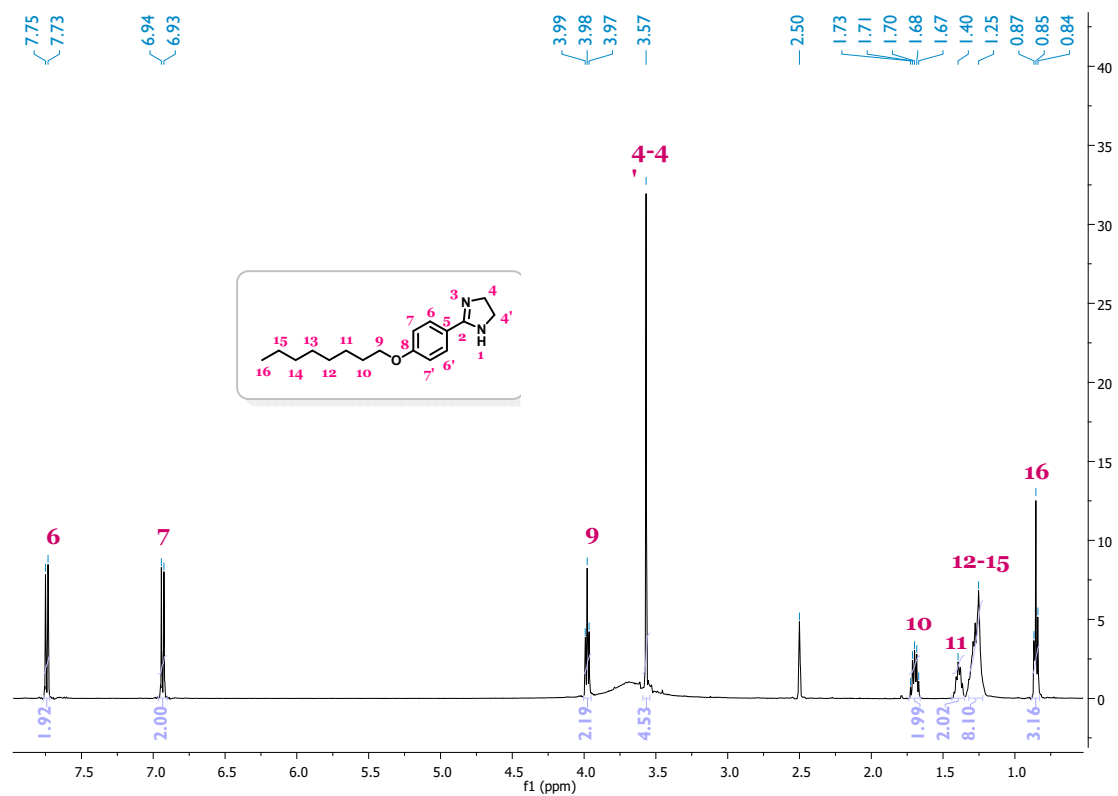

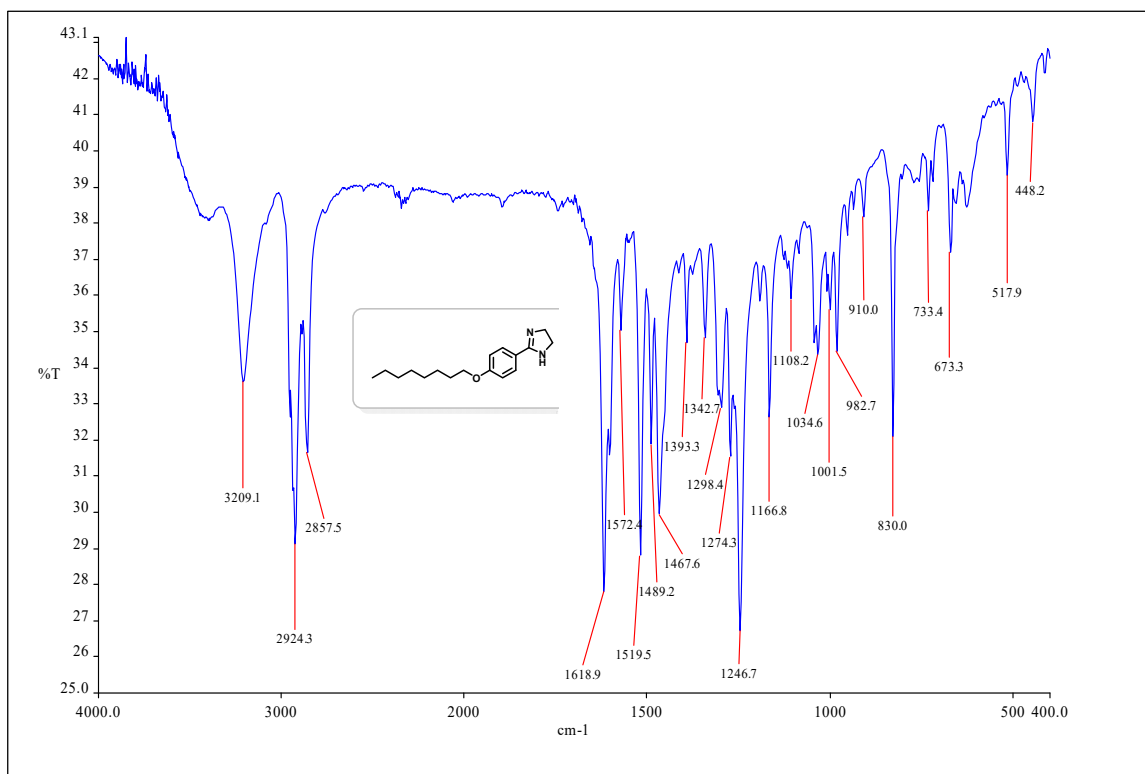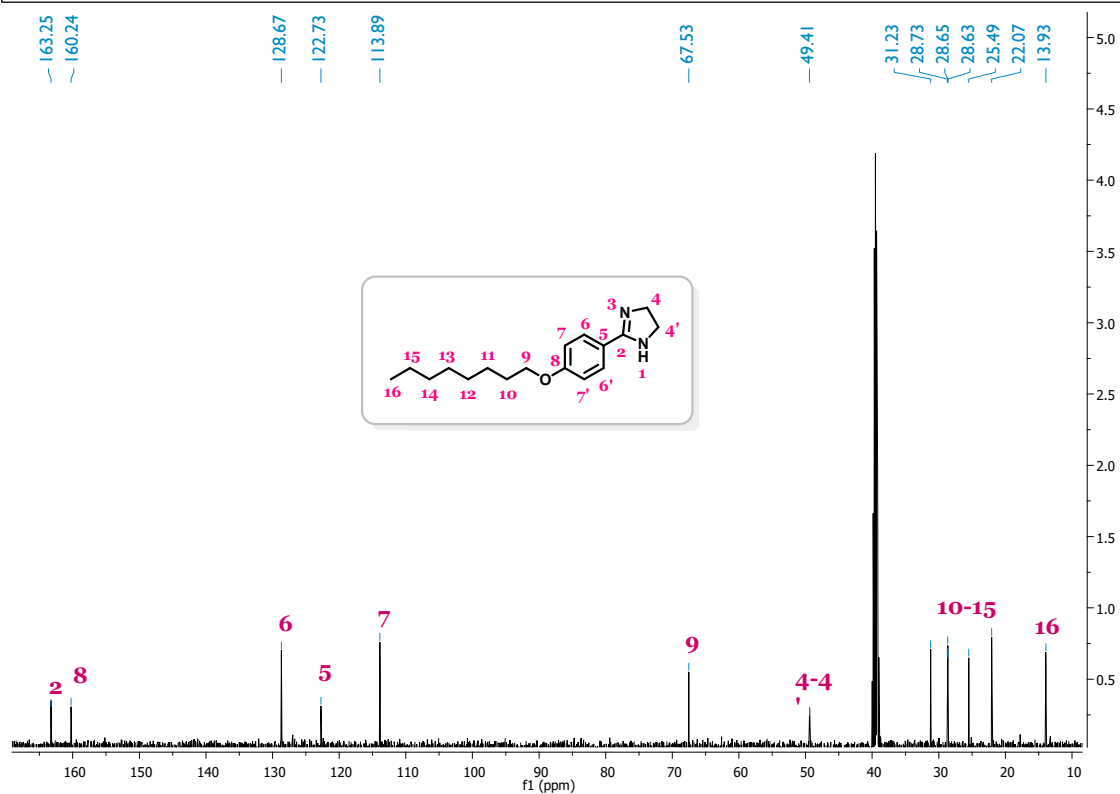

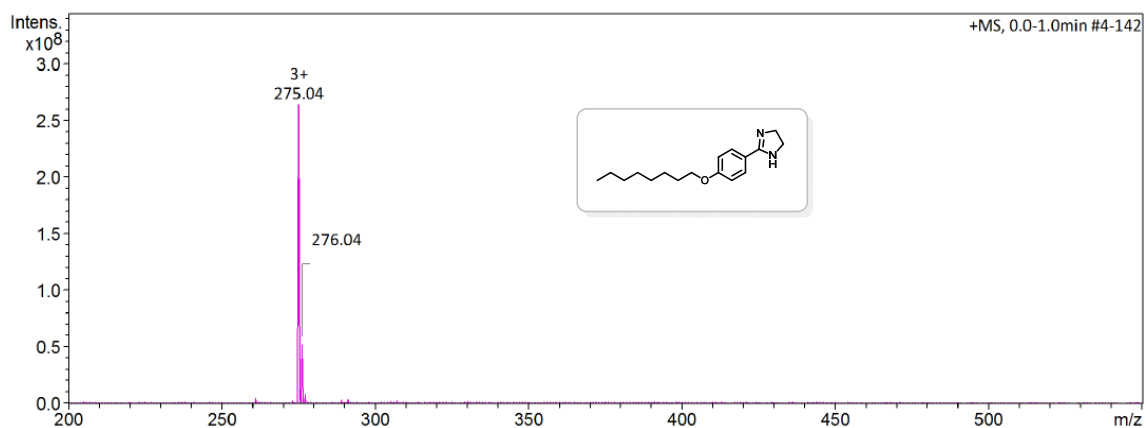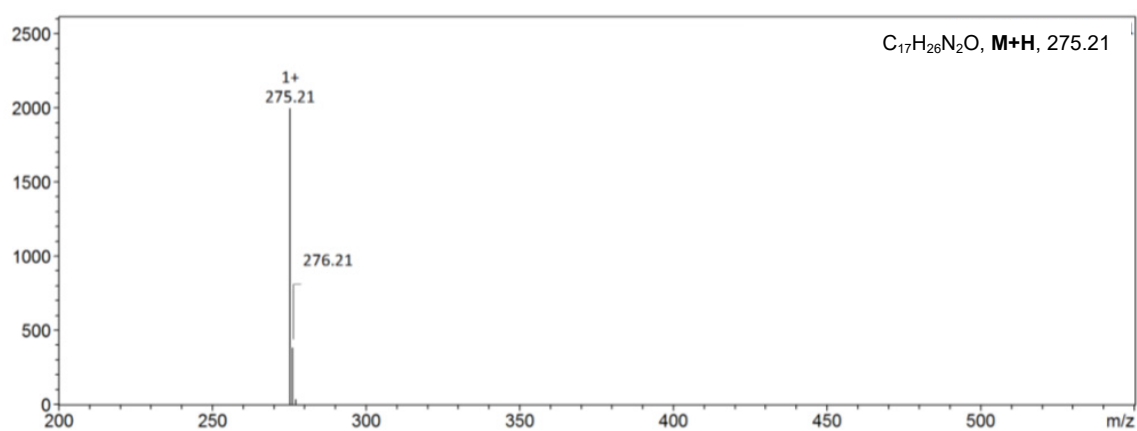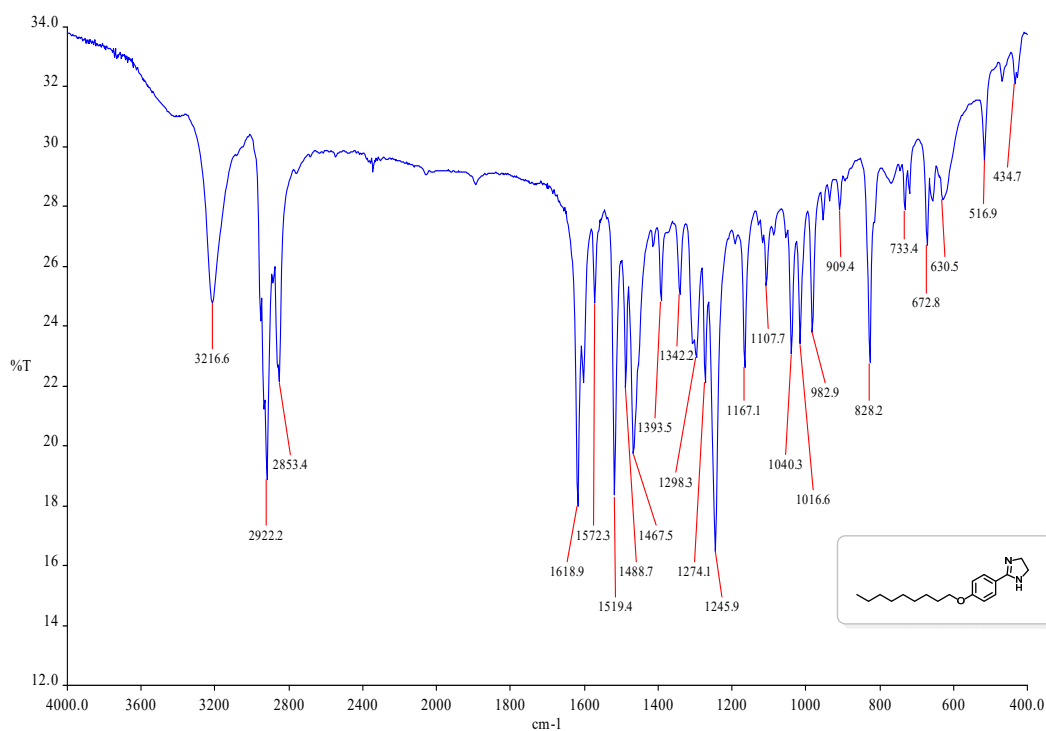

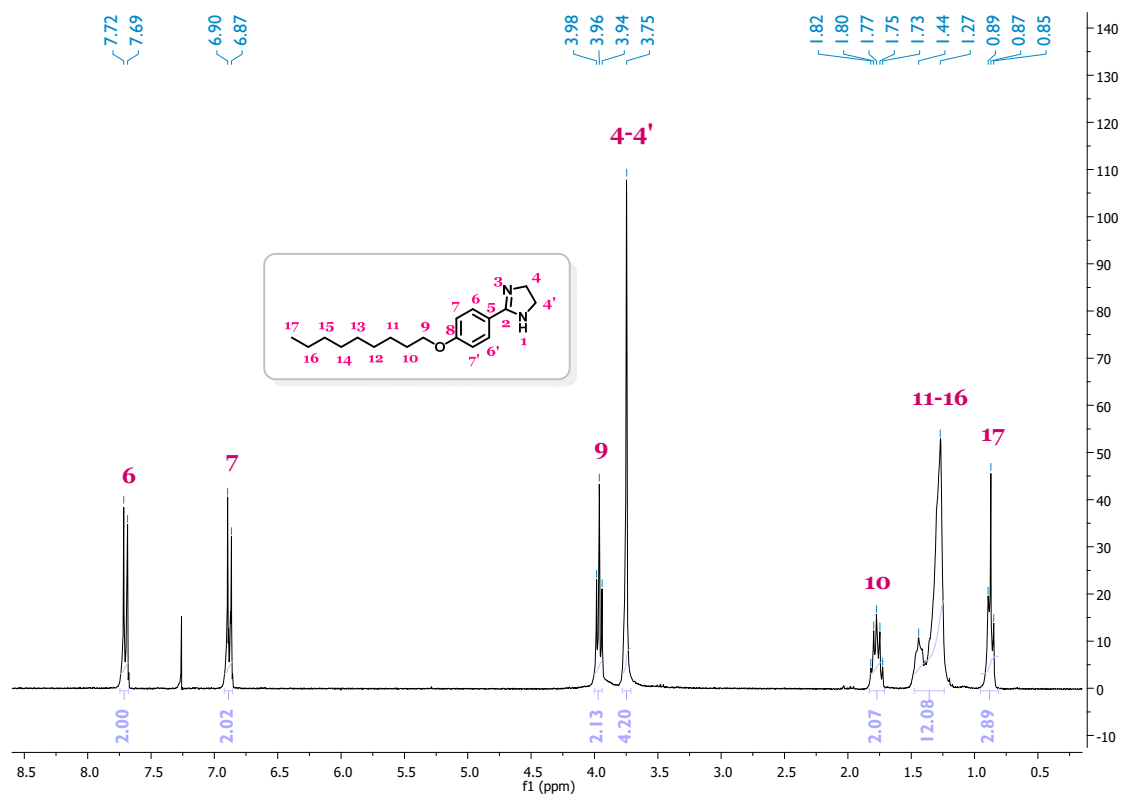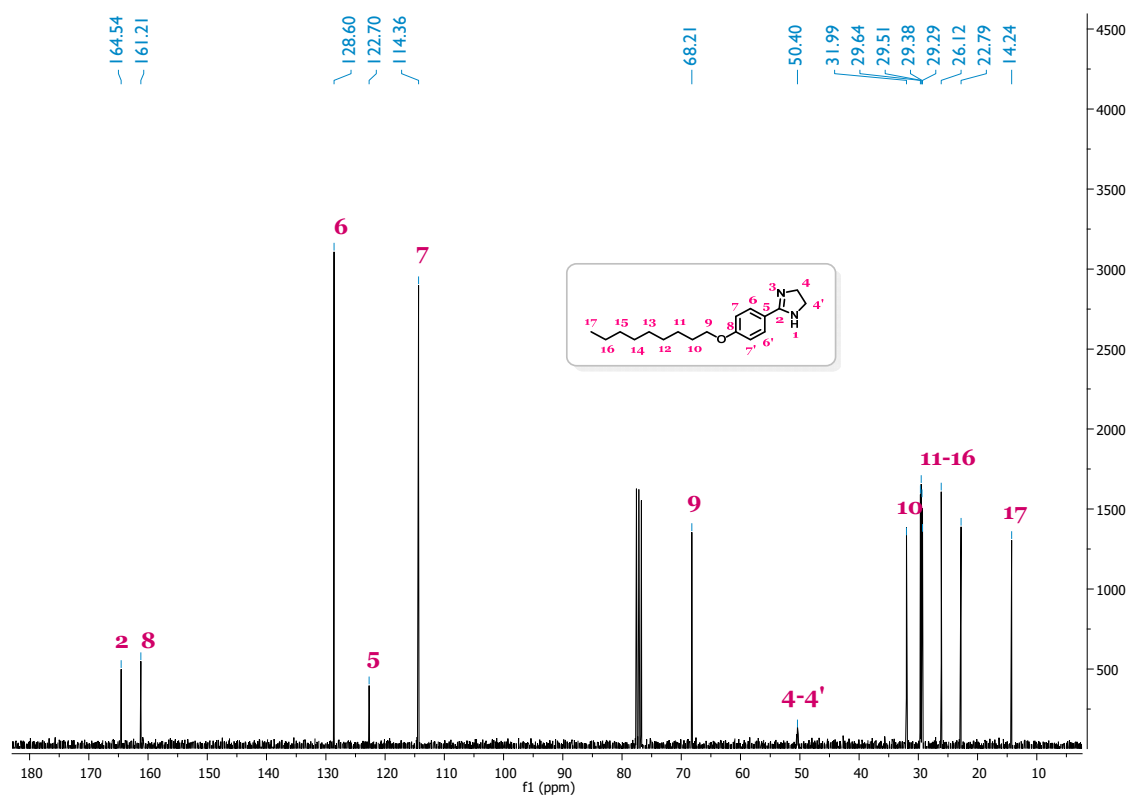

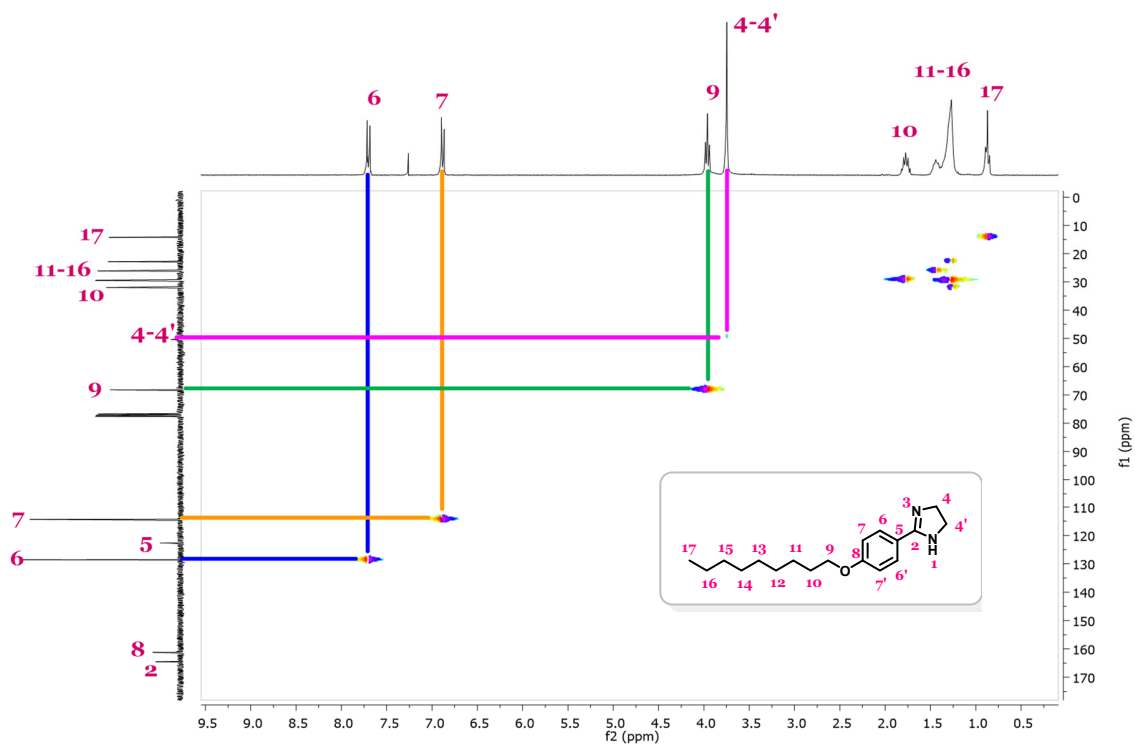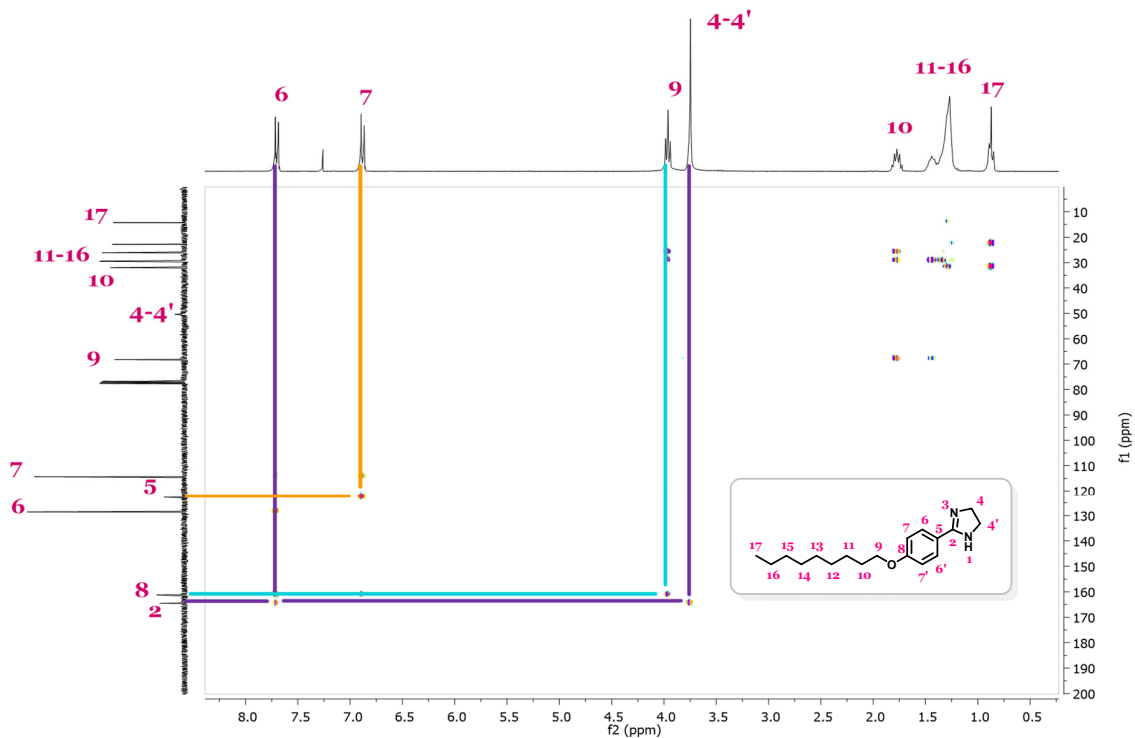

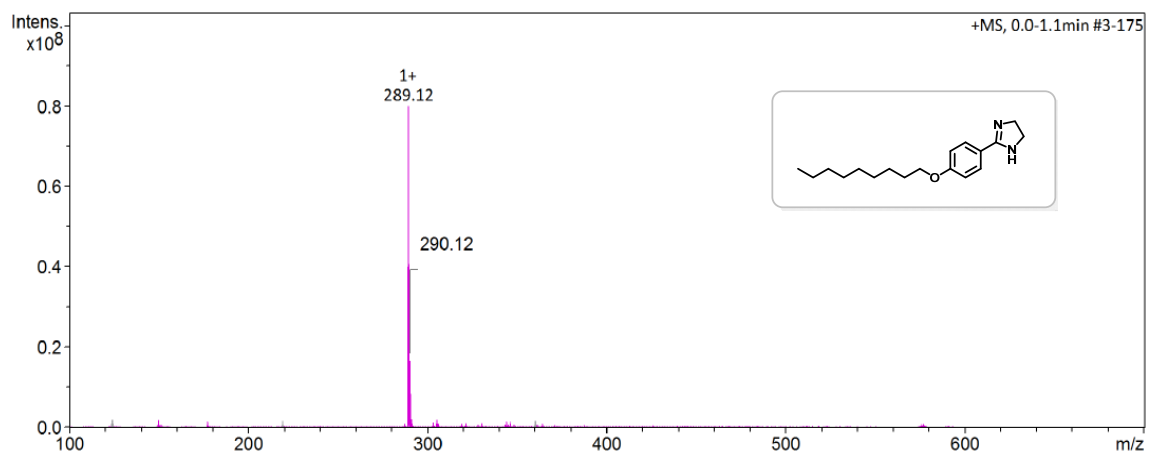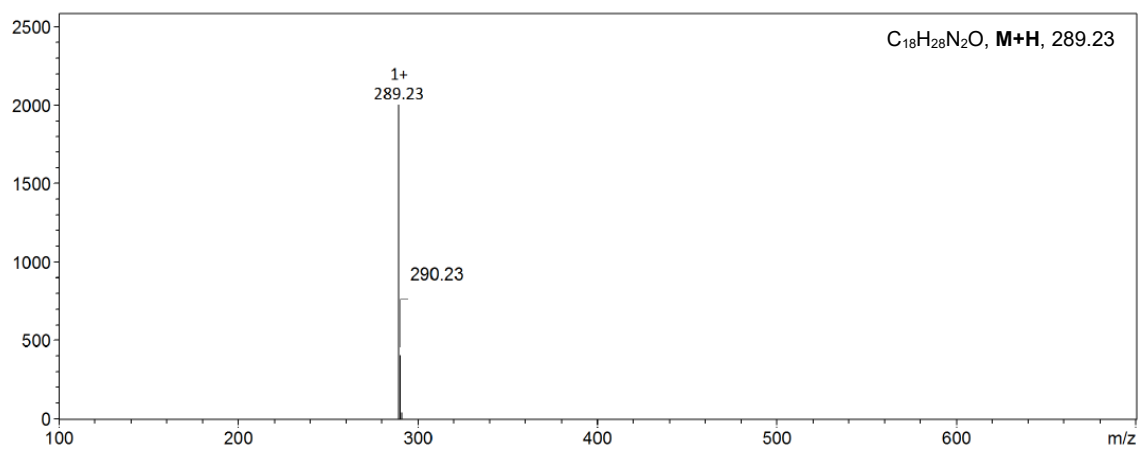

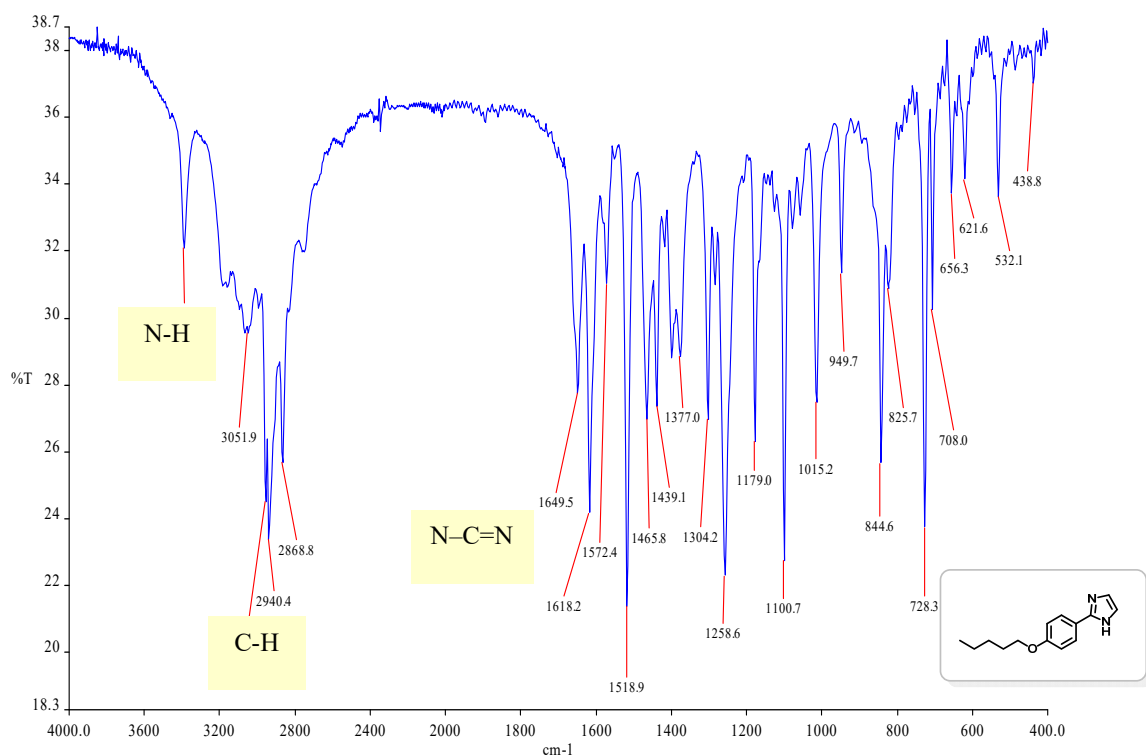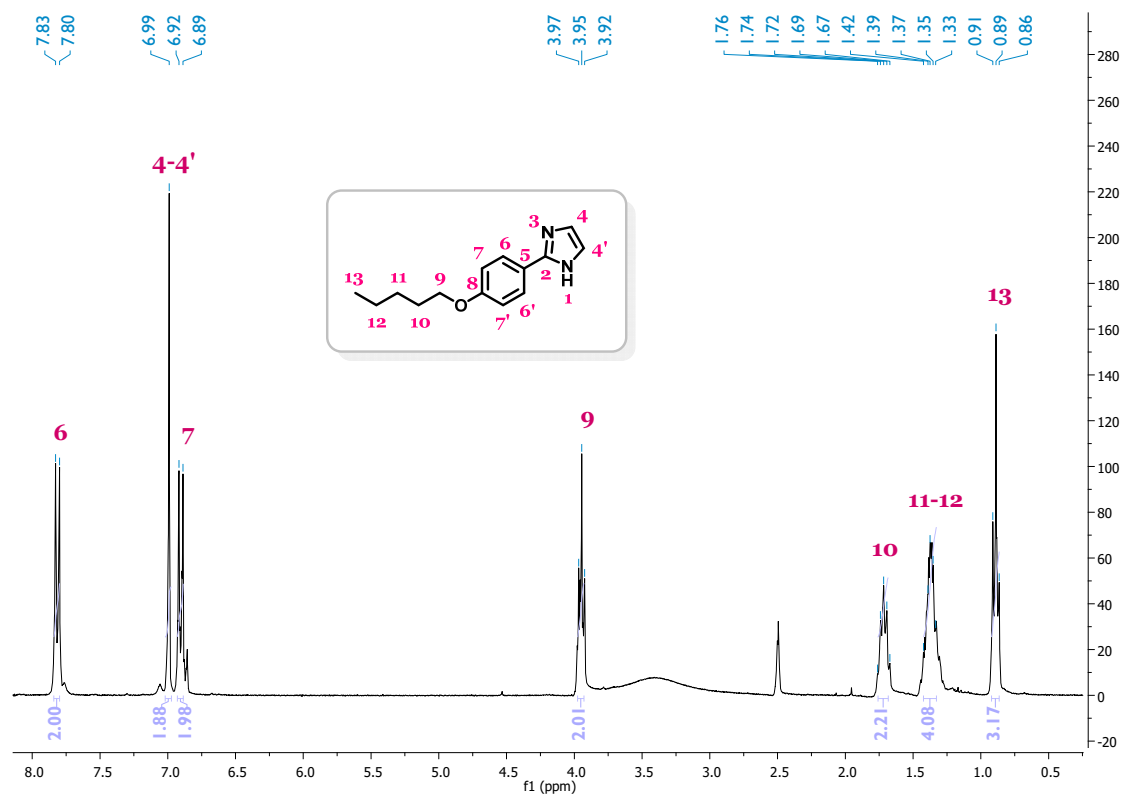

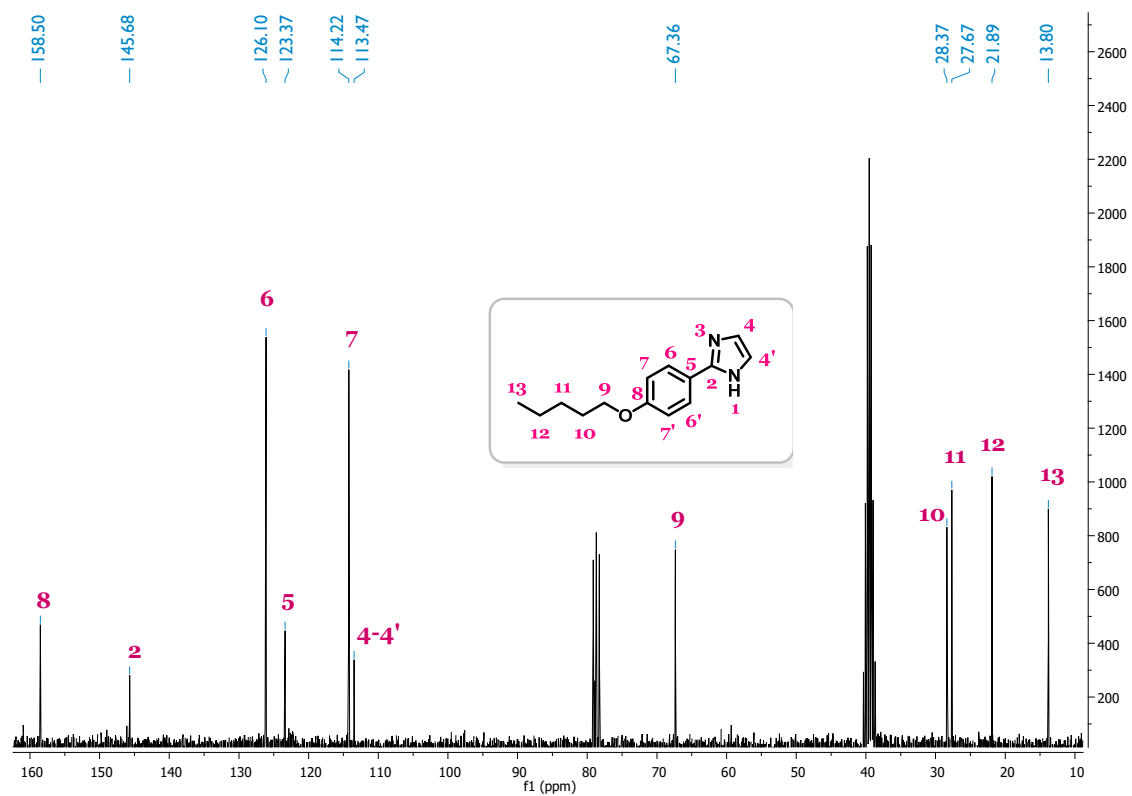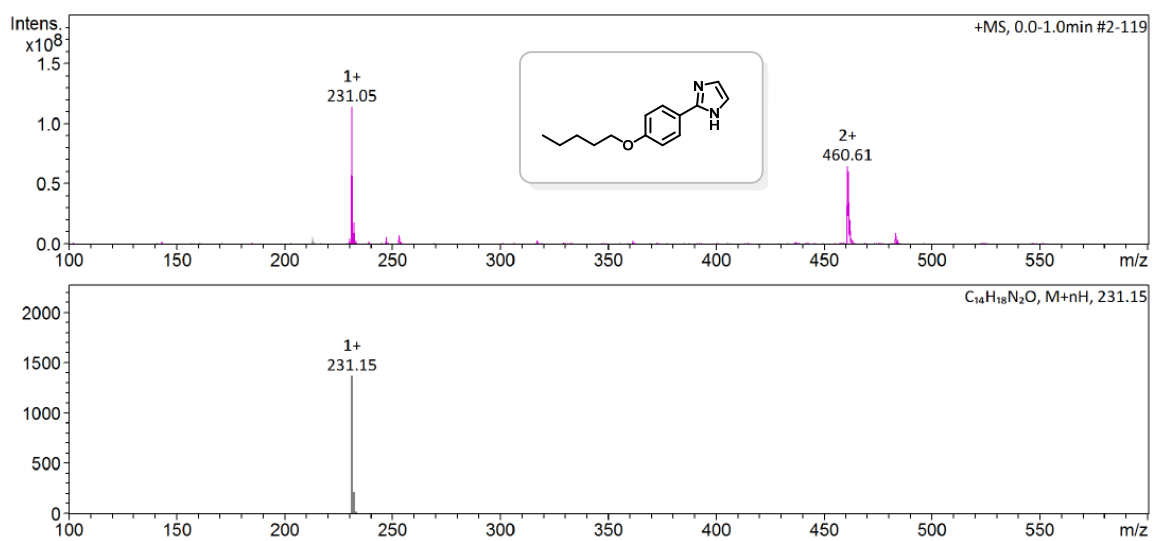

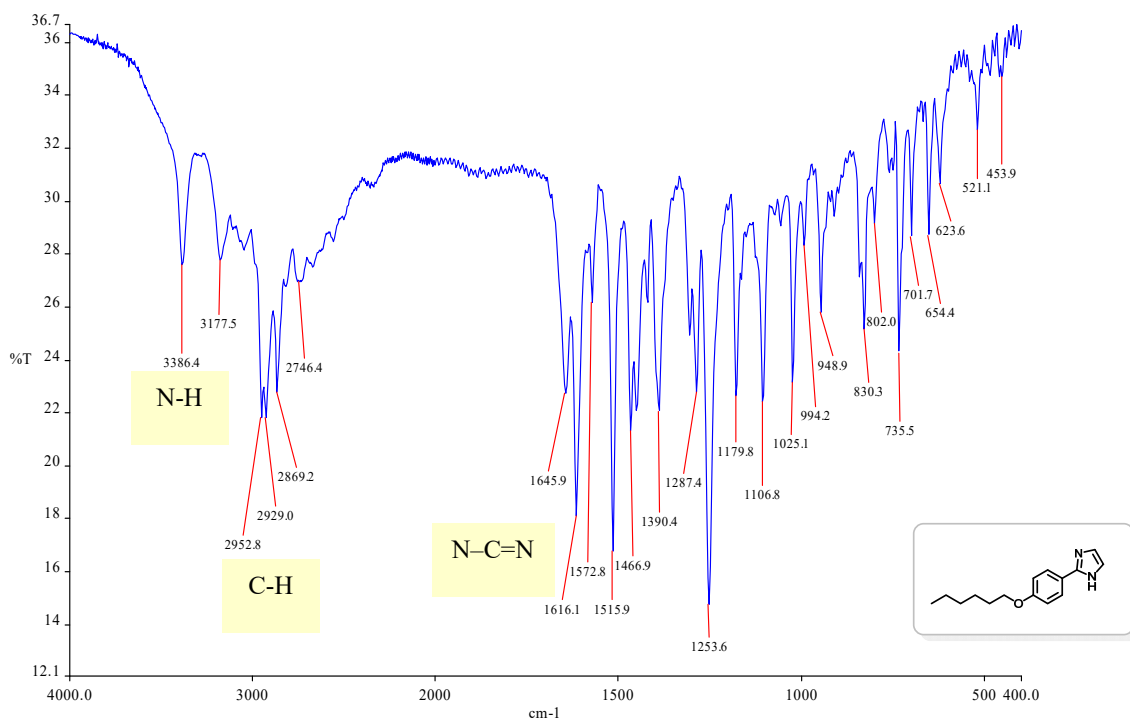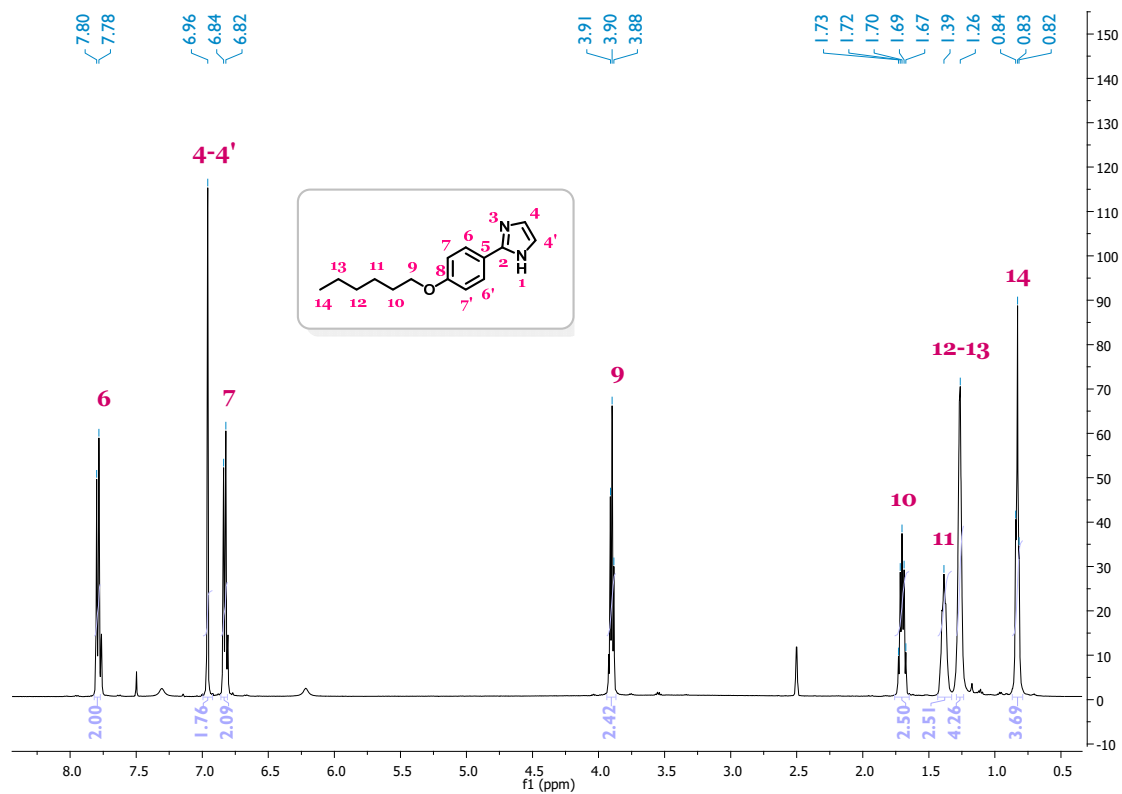

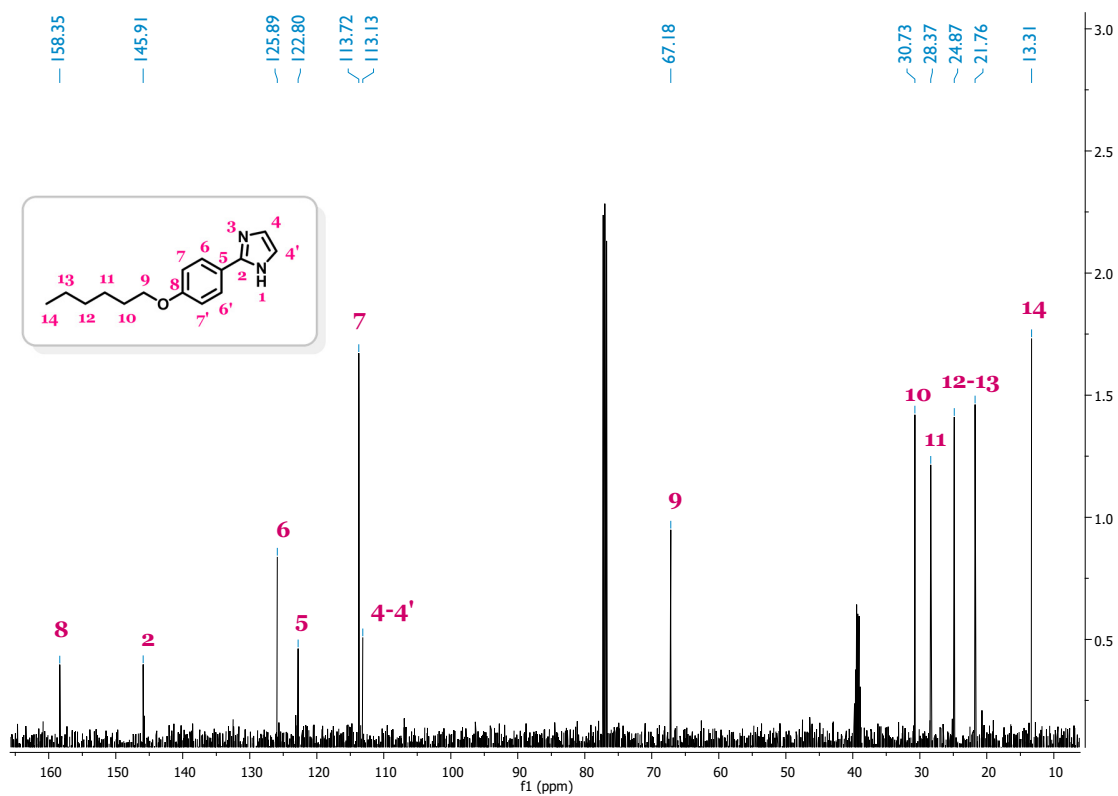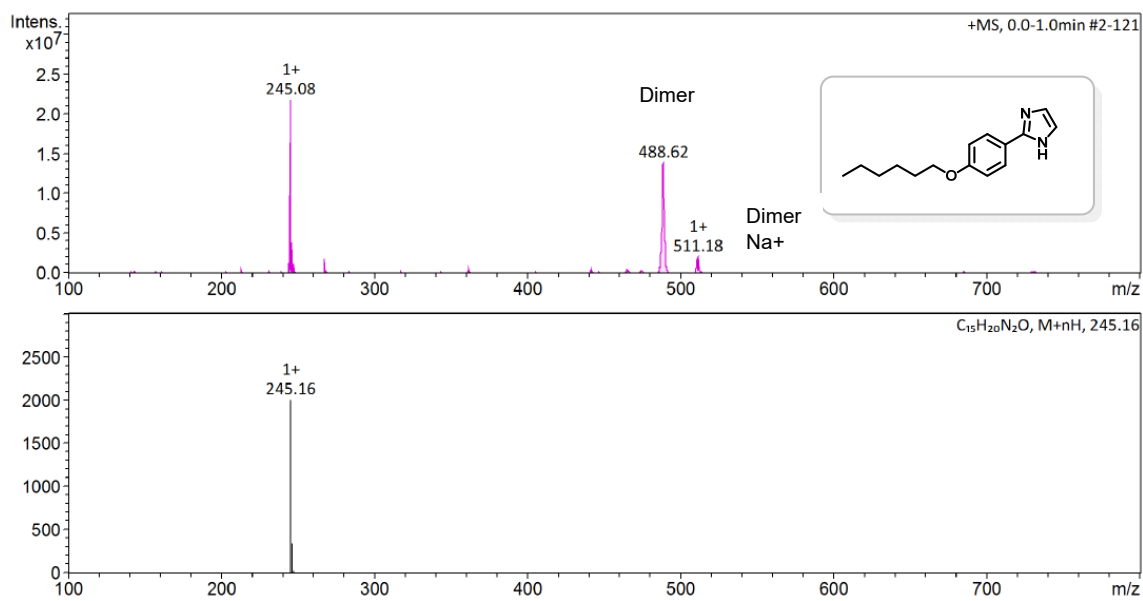

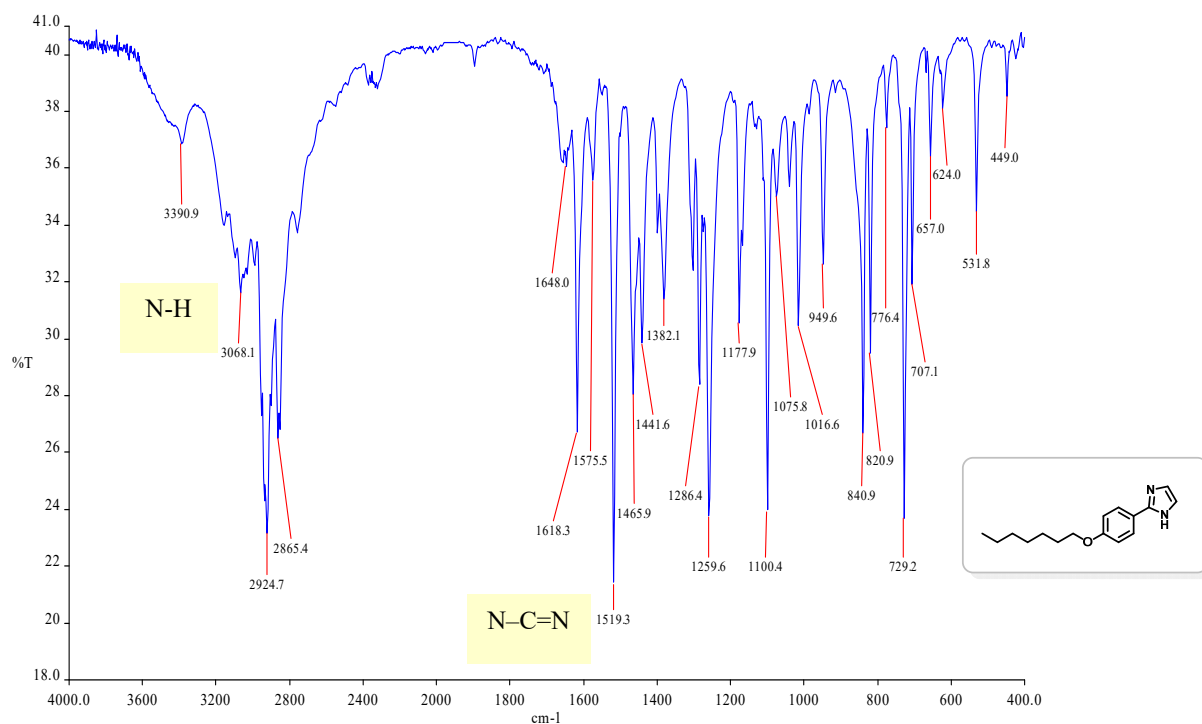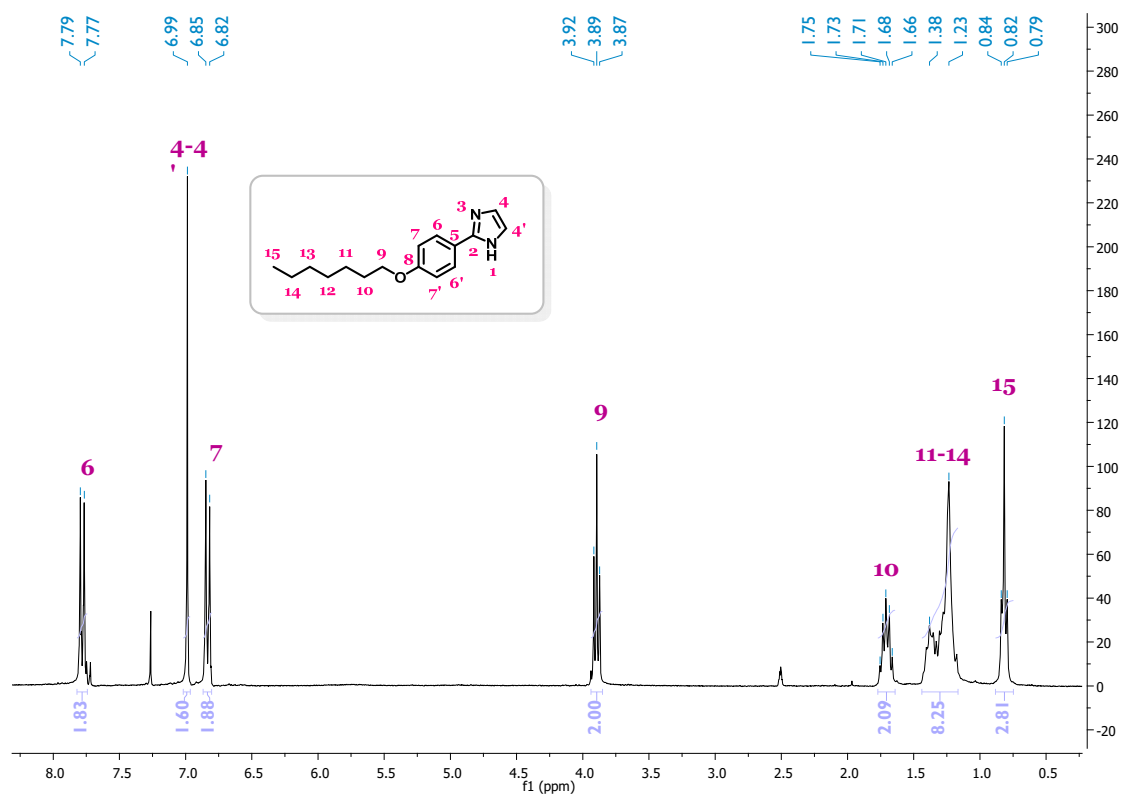

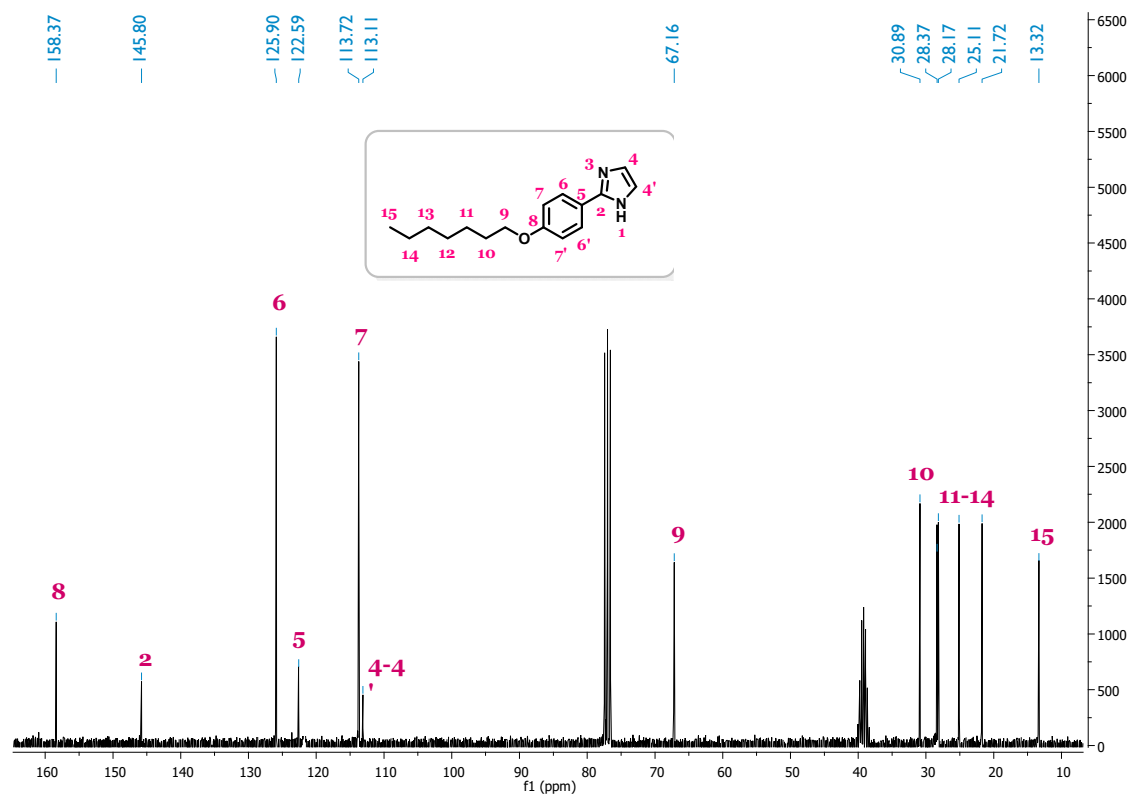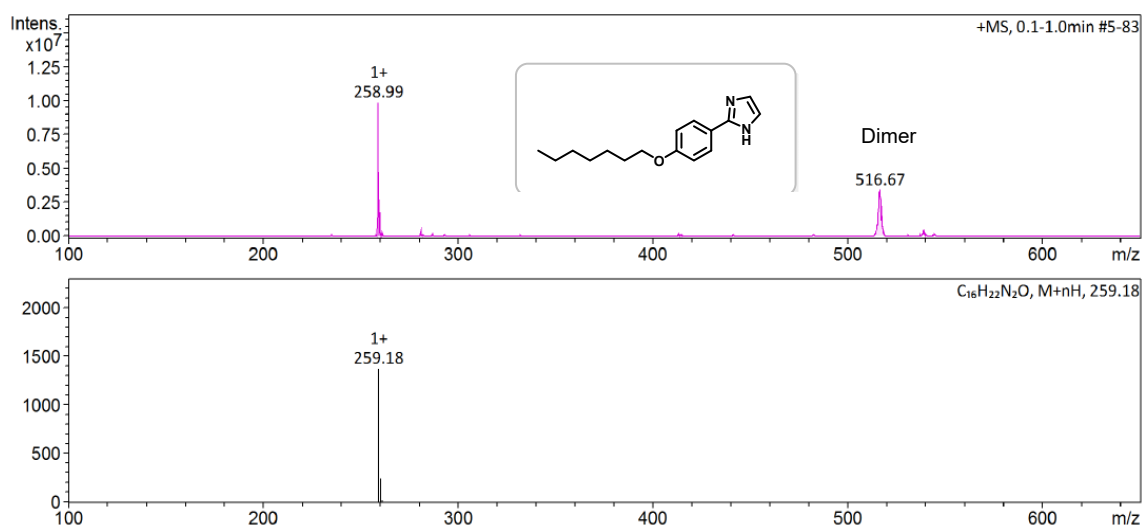

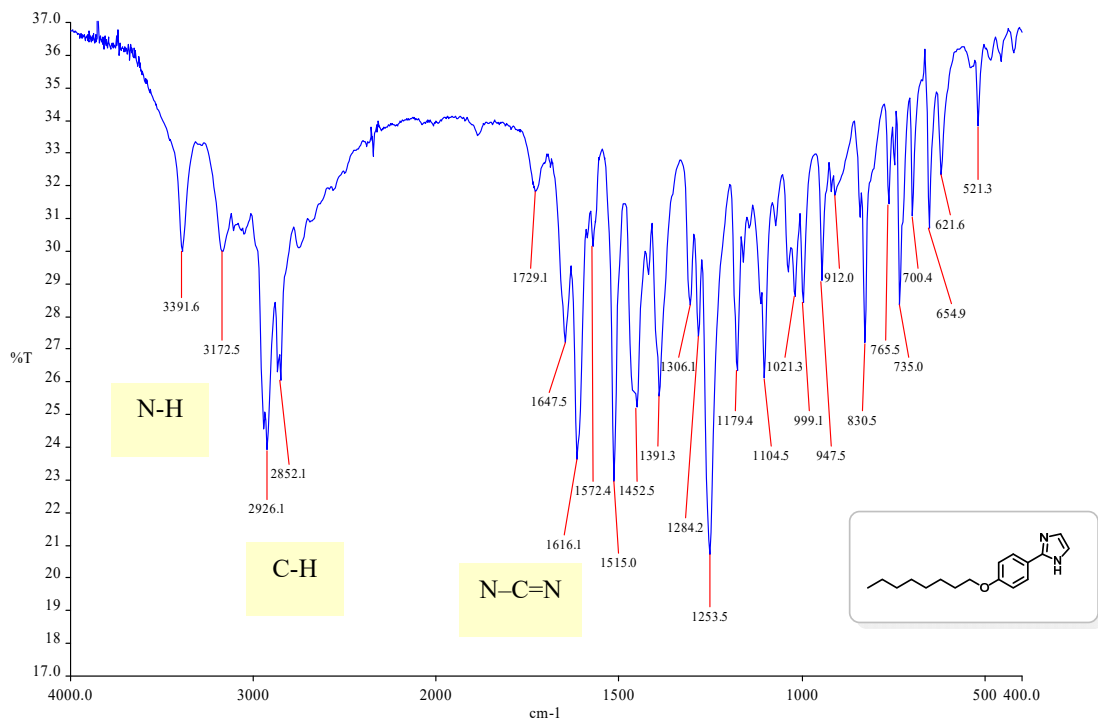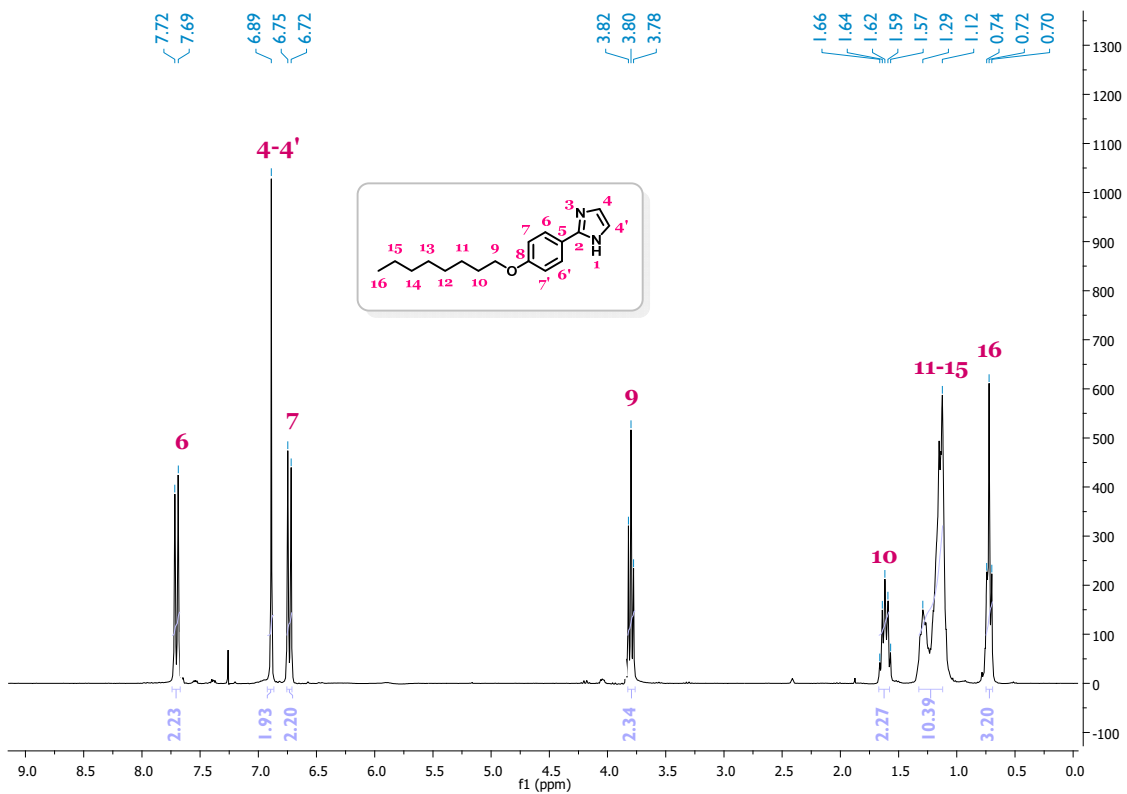

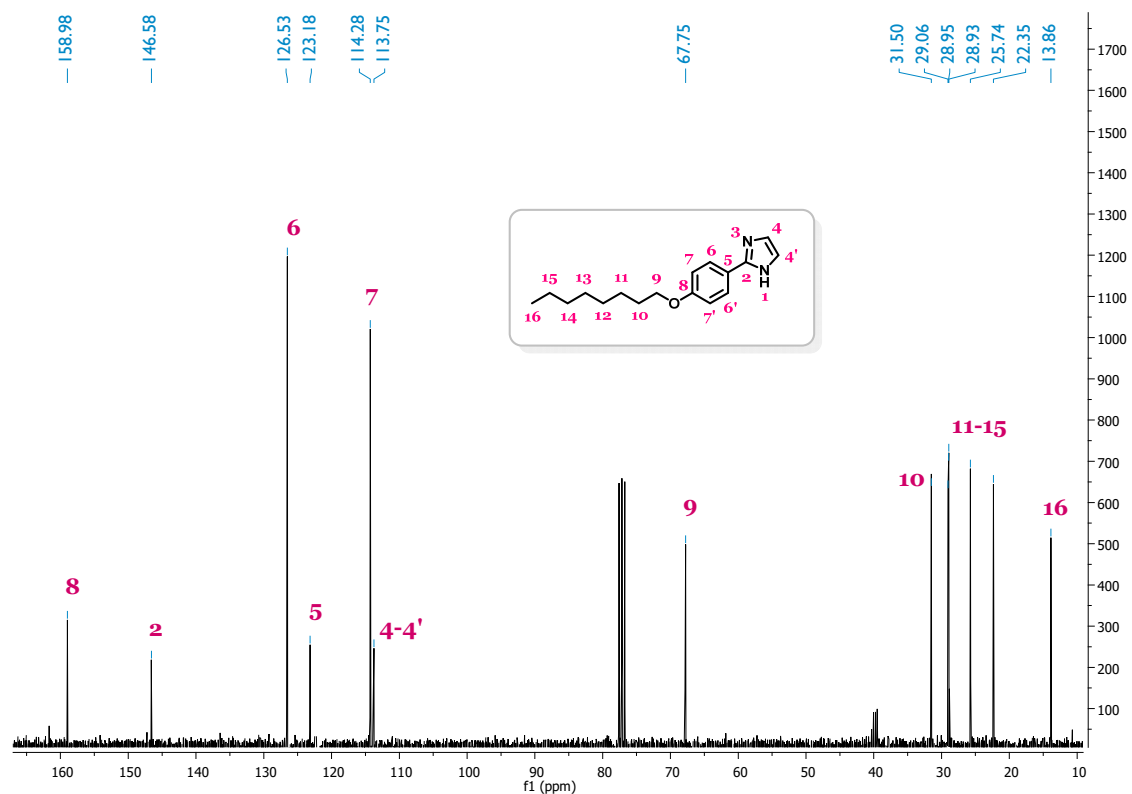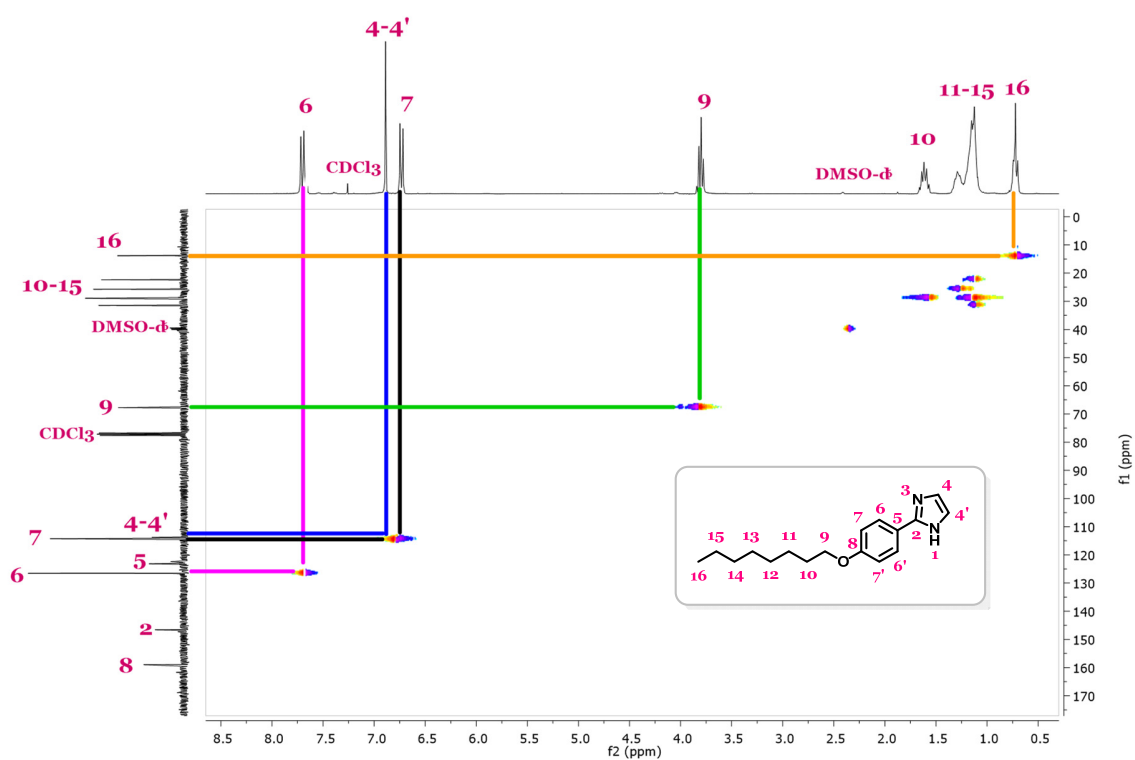

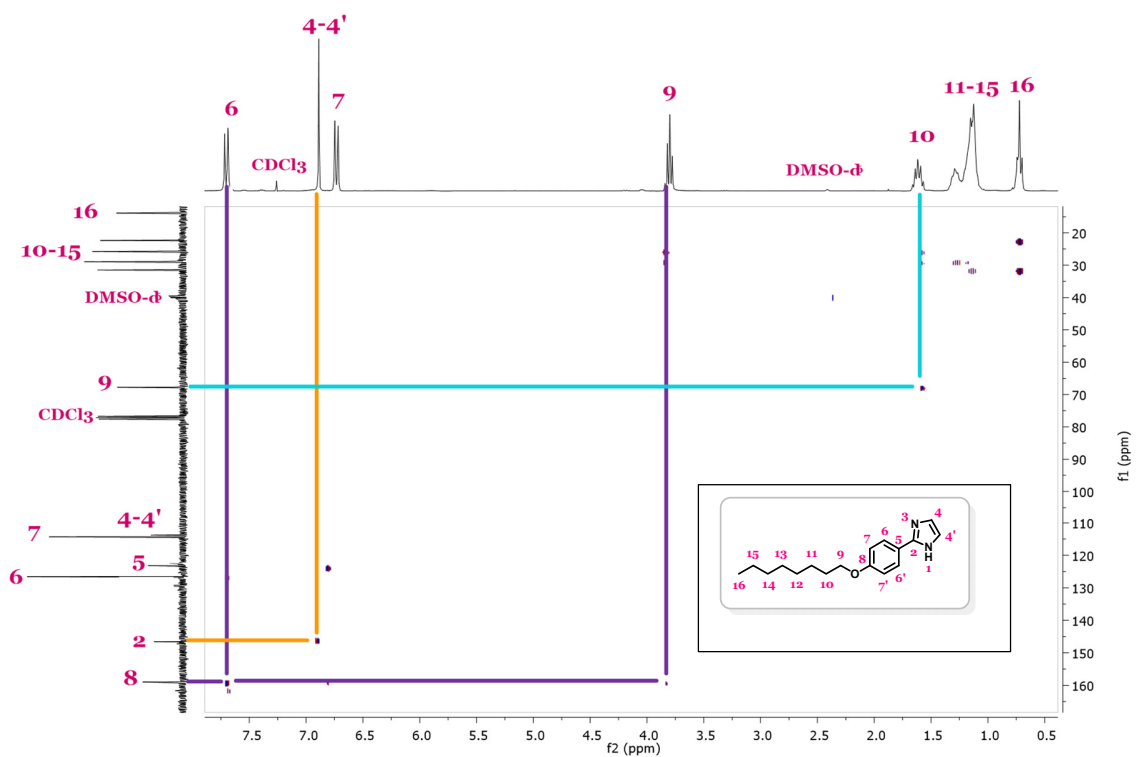

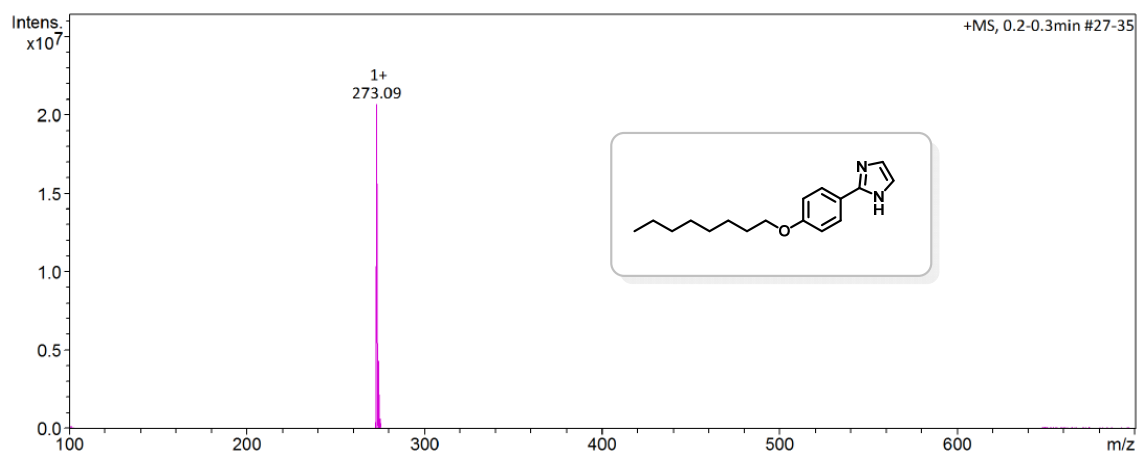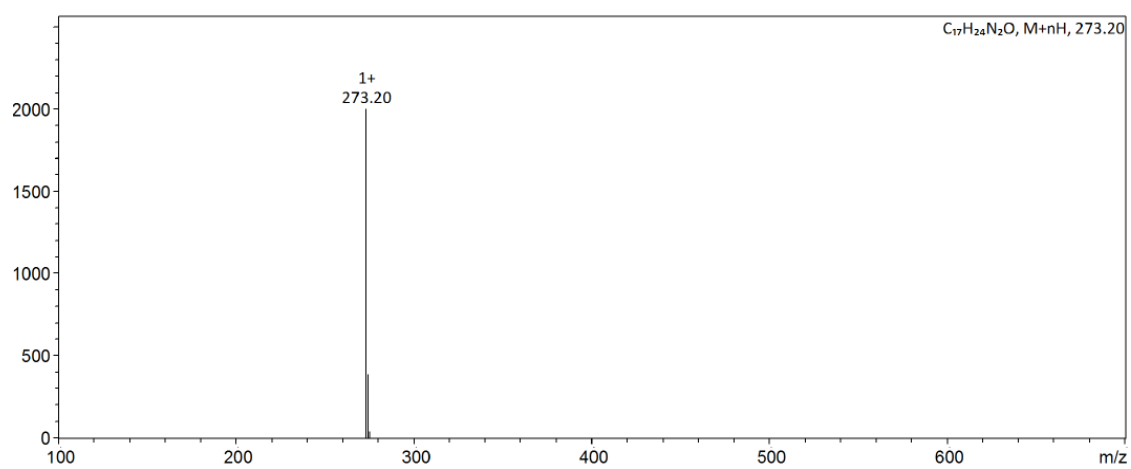

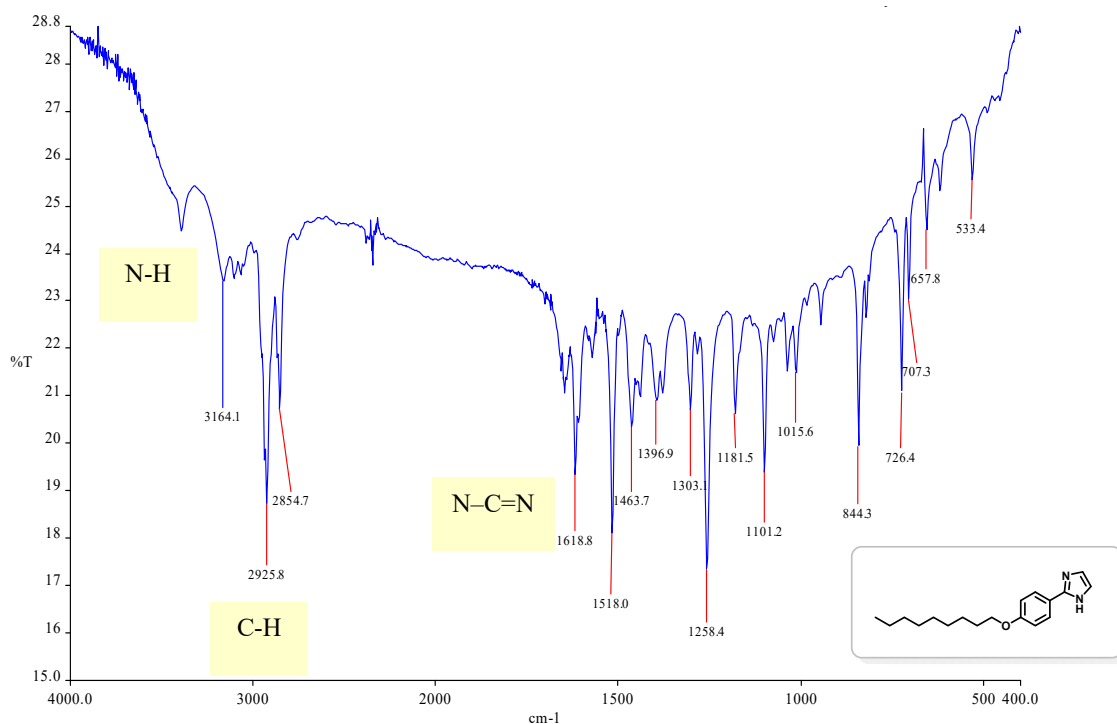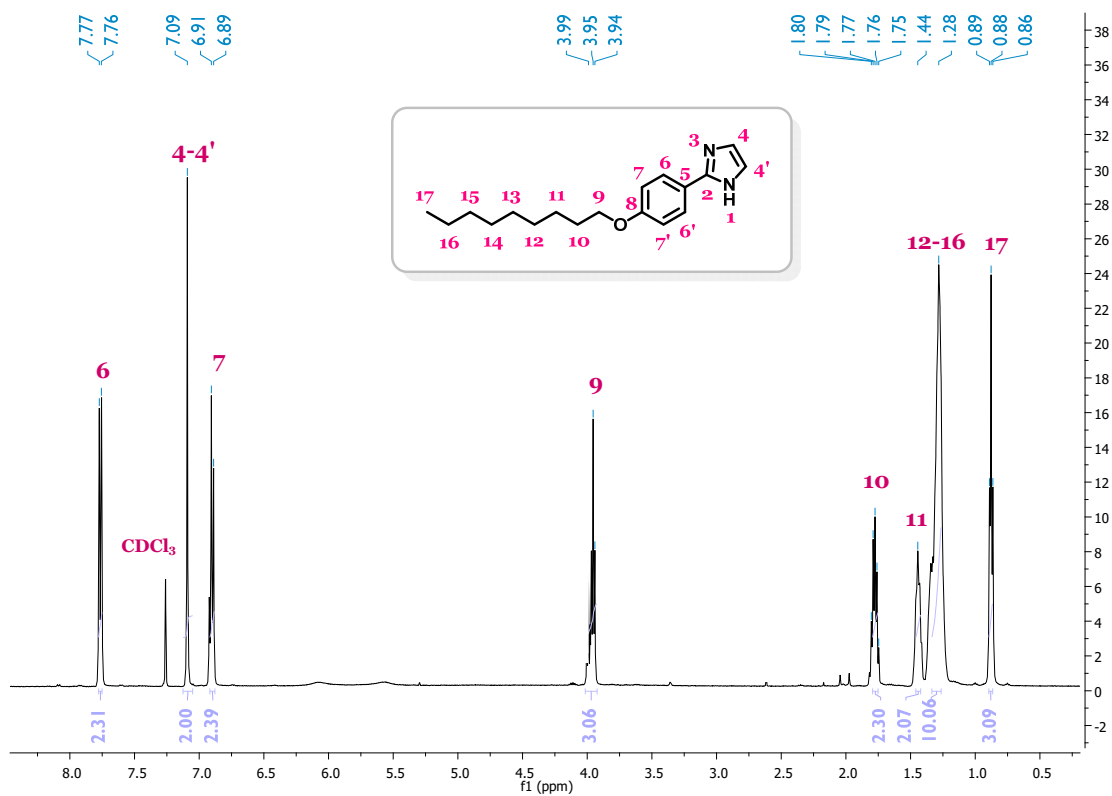

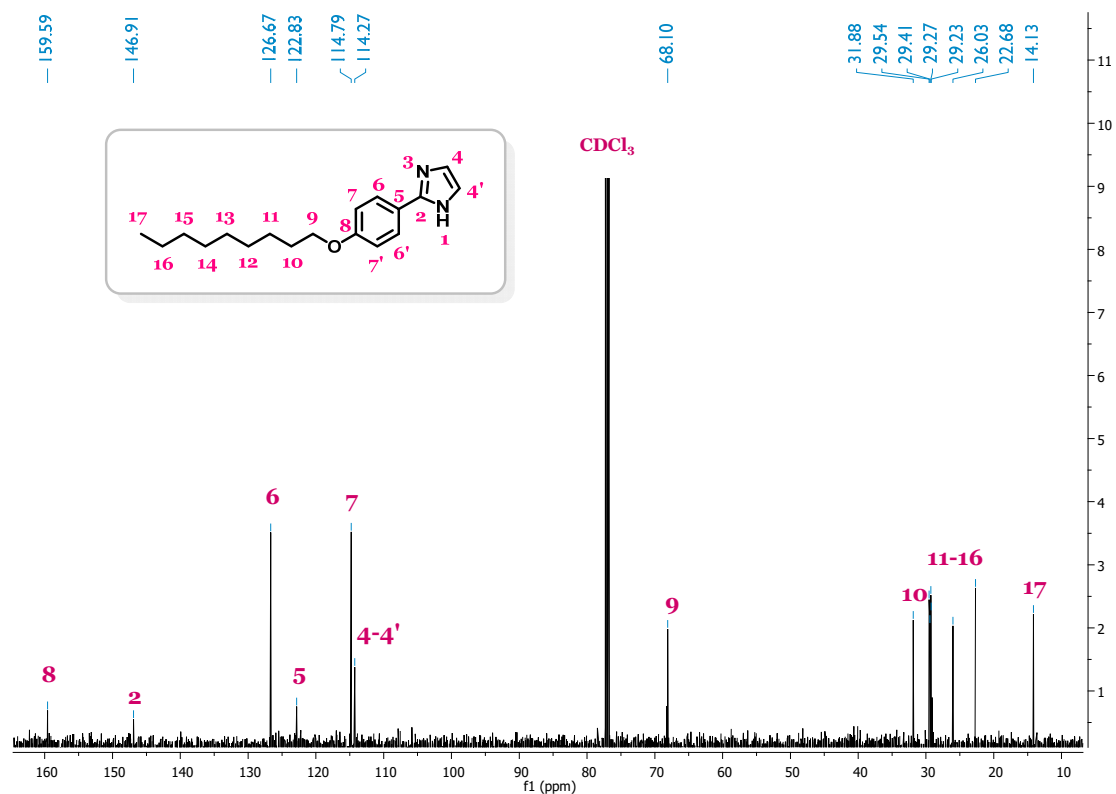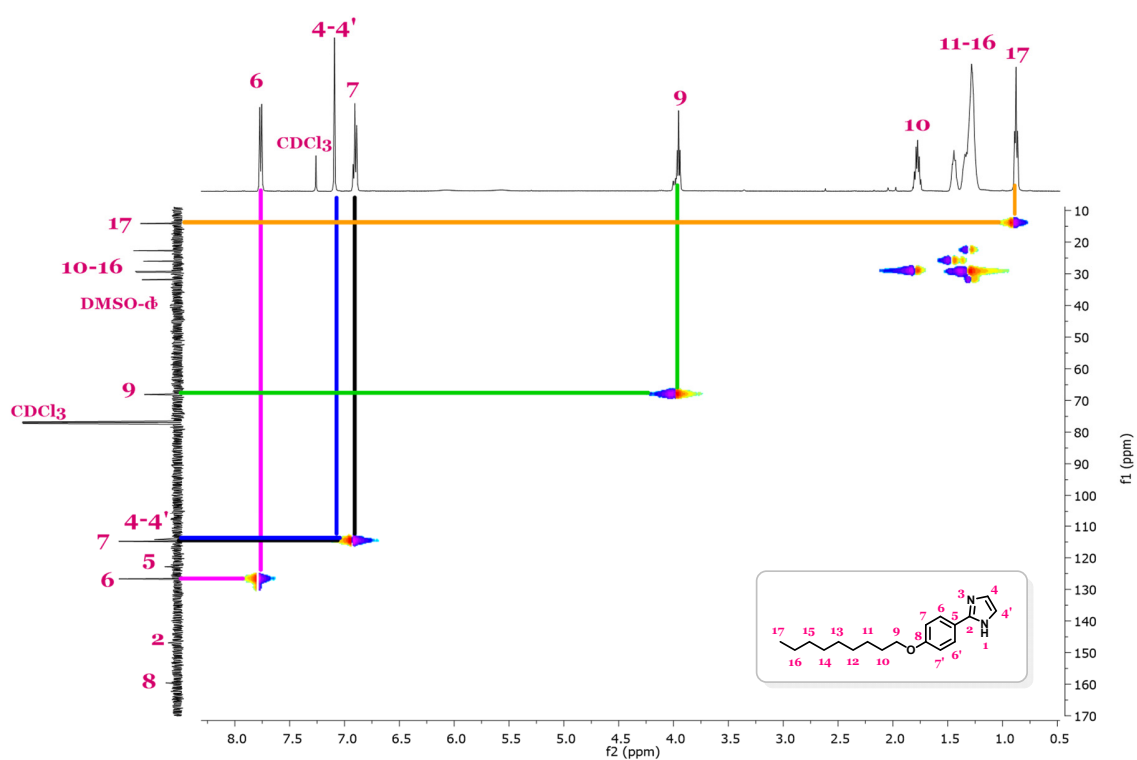

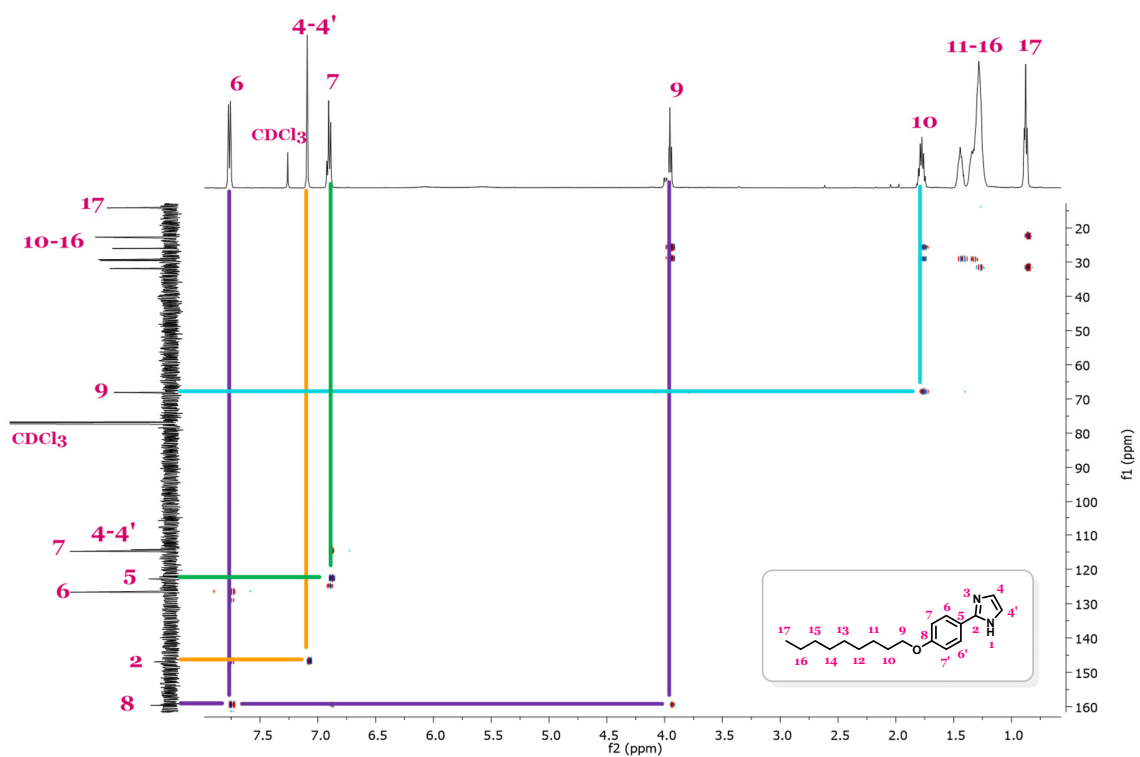

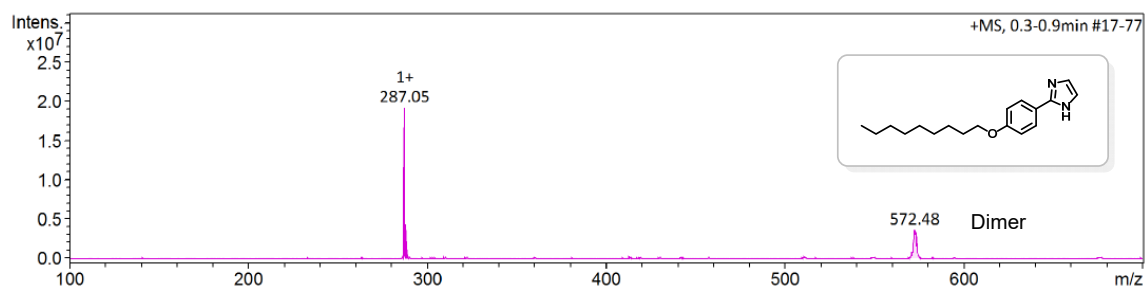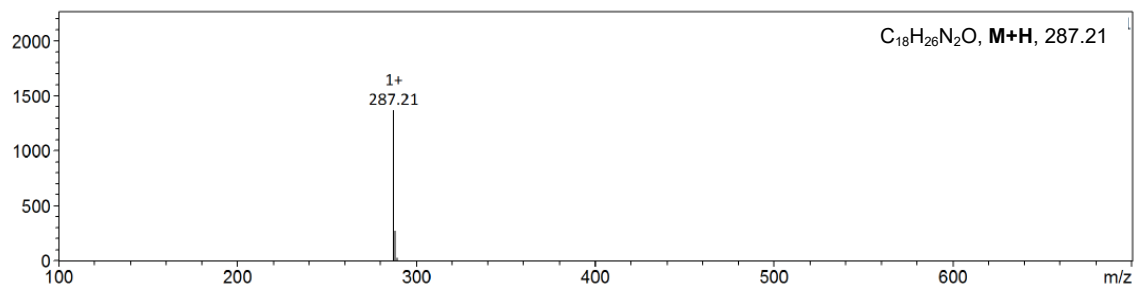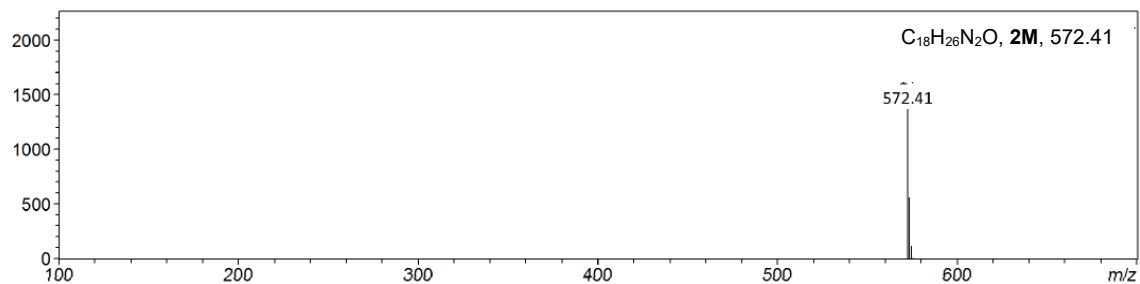

Supplement: Supplementary file 1 [file ijms-25-03673-s001.zip › ijms-2900811-supplementary.pdf]
